# 2015 AAPM Spring Clinical Meeting ‐ Abstracts

**DOI:** 10.1120/jacmp.v16i3.5634

**Published:** 2015-05-08

SATURDAY, MARCH 7

Best Poster Competition Exhibit Hall

**PO‐BPC‐Exhibit Hall‐01**

## Staff Radiation Protection for Oropharyngeal Motility Studies

X Jiang,^*^ A Dachman, I Reiser, K Little, ZF Lu

*The University of Chicago Medicine, Chicago, IL*

### Purpose

A potential radiation safety concern during oropharyngeal motility (OPM) studies is the radiation dose to the speech pathologist who usually stands close to the seated patient and receives relatively high scattered radiation. In this study, we report our efforts at measuring the scattered radiation distribution pattern and communicating the findings using easy visual aids, so that the speech pathologist is better informed of the locations of the high exposure areas and can adjust position during the procedure to reduce exposure.

### Methods

Scattered radiation distribution pattern was measured in two suites equipped with Siemens Luminos Agile fluoroscopy systems. An anthropomorphic phantom was used to simulate the patient. The study was performed in the upright position with standard OPM protocol. The scattered radiation exposure rate was measured using a survey meter around the phantom at 15° intervals and at 1.0 m, 1.5 m, and 2.0 m distances. The high exposure areas were identified and marked with warning tapes on the floor. The speech pathologists were informed of the results and advised to avoid the marked areas during beam‐on time.

### Results

Two areas with relatively high scatter exposure rate were identified. The first one (~50 mR/h at 1 m) is lateral to the patient and is caused by the gap between the bed and bedside lead drape. This is also the area where the speech pathologist tends to stand for easy access to the patient. The second area (~35 mR/h at 1 m) is behind the patient bed at ~30° from the X‐ray tube, which is caused by scattered radiation coming through the gap between the bedside shield and the X‐ray tube.

### Conclusion

High exposure areas during OPM studies were found to be limited to a few isolated locations and were system‐dependent. Marking these areas helps the operators easily recognize them and adjust their position accordingly.

**PO‐BPC‐Exhibit Hall‐02**

## Intermediate Dose Calculation During Optimization Improves Plan Quality for Lung IMRT and SBRT

S Gardner,^*^ A Doemer, B Miller, I Aref, M Ajlouni, B Movsas, I Chetty

*Henry Ford Health System, Detroit, MI*

### Purpose

To quantify the improvement in plan quality for lung IMRT plans utilizing intermediate dose calculation during IMRT optimization.

### Methods

For this study, 10 conventionally‐fractionated and 5 SBRT lung IMRT cases were analyzed. For comparison, the optimization was repeated (using same constraints) with intermediate dose calculation. Plan metrics for conventional plans were: conformity index (CI), PTV D(99%), and PTV D(10%). For SBRT plans: CI, PTV D(99%), Ratio of 50% isodose volume to PTV volume (50% Ratio), and the volume of tissue receiving the prescription dose outside the PTV (Vol. Rx‐Outside). All dose values were scaled as a percentage of the prescription dose; all volumes are given in cc. For each case, the original plan (‘Original’) was compared to the plan with intermediate dose calculation (‘Optimized’).

### Results

The intermediate dose calculation resulted in improvements in all plan quality metrics. For conventional IMRT lung cases, the mean CI was 1.21 (Original) and 1.06 (Optimized) [p<0.005]; the mean D(99%) was 96.8% (Original) and 98.0% (Optimized) [p<0.005]; the mean D(10%) was 109.3% (Original) and 104.0% (Optimized) [p<0.005]. For SBRT cases, the mean CI was 1.26 (Original) and 1.07 (Optimized) [p=0.010]; the mean 50% Ratio was 6.32 (Original) and 6.02 (Optimized) [p=0.019]; the mean D(99%) was 96.8% (Original) and 98.0% (Optimized) [p=0.016]; the mean Vol. Rx‐Outside was 4.04 cc (Original) and 0.82 cc (Optimized) [p<0.009].

### Conclusion

For optimized plans, the most substantial improvements were observed in PTV dose conformity and uniformity. Pencil beam‐based dose calculation provides inaccurate results when used for beamlet calculations. Optimization using AAA produces a more accurate dose distribution and drives the optimizer based on a different intermediate solution. Therefore, use of intermediate dose calculation in lung IMRT plan optimization has the potential to simultaneously improve planning efficiency and plan quality.

Varian holds research agreements with Varian Medical Systems (Palo Alto, CA).

**PO‐BPC‐Exhibit Hall‐03**

## Correlating Image Quality and Reduced Radiation Exposure From Low‐Dose CT Scanning in a Lung Cancer Screening Program

I Lipnharski,^*^ A Mench, C Carranza, R Lamoureux, S Gyapong, L Rill, T Mohammed, M Arreola

*University of Florida, Gainesville, FL*

### Purpose

To correlate acceptable image quality and reduced radiation doses by employing iterative reconstruction and manipulating exam parameters in low‐dose computed tomography (LDCT) for routine lung cancer screening.

### Methods

Three postmortem subjects were scanned on a commercial 320‐slice CT scanner using a lung cancer screening protocol, reconstructed with an iterative algorithm (AIDR‐3D). The minimum tube current was decreased from a default setting of 100 mA to 30 mA, with tube current modulation employed. Further dose savings were attained by increasing noise target levels from a standard deviation of 12.5 to 17.5, 20, and 25. The dose length product (DLP) reported by the scanner was recorded for each scan. Organ doses were directly measured in one of the postmortem subjects using *in vivo* dosimetry methods utilizing optically stimulated luminescent dosimeters. Eleven radiologists were recruited to perform a blinded observer study, grading the images with a score of (1) for nondiagnostic, (2) for suboptimal, or (3) for diagnostically acceptable.

### Results

With the reduced tube current employed, increasing the noise tolerance index achieved significant dose savings of 40%, 49%, and 66%, for standard deviations of 17.5, 20, and 25, respectively. The majority of readers found all images to be acceptable. It was not until very high dose savings of 66% that one of the readers found the image to be unacceptable.

### Conclusion

With the nationwide acceptance of the lung cancer LDCT initiative, it is imperative that these scans be performed using very low dose protocols. Due to the high likelihood of repeat scans and elevated cumulative radiation dose, the consideration of this at‐risk population must focus on low dose. Together with AIDR‐3D, which offers dose savings in the range of 20%–40%, employing reduced tube current and increased noise tolerance can offer substantial dose reduction of 75%, while still maintaining acceptable image quality.

**PO‐BPC‐Exhibit Hall‐04**

## Commissioning of Breath Hold for Motion Management of Stereotactic Body Radiation Therapy Using a Six Degrees‐of‐Freedom Motion Platform and Three‐Dimensional Diode Array

J James,^*^ A Cetnar, B Wang

*University of Louisville, Louisville, KY*

### Purpose

The motivation for using breath‐hold as a motion management technique for SBRT instead of respiratory gating is that it allows you to acquire a pretreatment cone‐beam CT for direct setup verification. In order to commission breath hold for motion management of SBRT, a six degrees‐of‐freedom motion platform and three‐dimensional diode array were used.

### Methods

Respiratory waveforms that include a breath‐hold period were created for varying levels of deep inspiration breath hold (DIBH) and breath hold at end of expiration. These waveforms were then used in conjunction with the ScandiDos HexaMotion motion platform to create planning CTs of the ScandiDos Delta^4^ three‐dimensional diode array in the breath‐hold position. VMAT plans were optimized for each breath‐hold CT and delivered to the Delta^4^ phantom using the Varian TrueBeam gating system in breath‐hold mode. The expected dose was compared to the delivered dose using a gamma analysis. In addition, the breath‐hold gating limits were investigated to determine how wide the gating range can be with acceptable dosimetric accuracy.

### Results

Each breath‐hold treatment delivery had a gamma passing rate within our clinical criteria, ranging from 99.7% to 100.0% for 3%/3 mm and 96.3% to 99.0% for 2%/2 mm. When allowing the breath‐hold waveform to drift between gating limits or to hold steady at one extreme of the gating range, the gamma passing rates decreased as expected. The most significant decrease occurred when increasing the gating range from 4 mm to 5 mm. The 3%/3 mm and 2%/2 mm gamma pass rates decreased from 99.7% to 95.7% and from 95.1% to 72.9%, respectively.

### Conclusion

Breath hold was shown to be an effective and dosimetrically accurate way to provide motion management for SBRT using the TrueBeam gating system. We recommend the use of a 4 mm gating window when implementing breath hold in the clinic, to provide the proper balance between treatment efficiency and dosimetric accuracy.

**PO‐BPC‐Exhibit Hall‐05**

## Ultrasound Guided Radiation Therapy of the Canine Urinary Bladder Using a Custom‐Designed Image Guidance Platform

J Sick,^1*^ N Rancilio,^2^ S Blake,^1^ H Heng,^2^ J Poulson,^1,2^ D Knapp,^2^ K Stantz^1^

*School of Health Sciences,^1^ Purdue University, West Lafayette, IN, College of Veterinary Medicine,^2^ Purdue University, West Lafayette, IN*

### Purpose

Radiation therapy (RT) for the treatment of transitional cell carcinoma (TCC) is technically difficult due to potentially large interfraction variations in bladder size, shape and position. The goal of this research is to develop real‐time, image‐guided radiotherapy techniques based on ultrasound instrumentation and methods. The proposed design is to combine US and photoacoustic (PA) molecular imaging in a single system that can be positioned on the radiation therapy treatment couch.

### Methods

We constructed the platform and calibrated it to the coordinate system of the linear accelerator couch. This was done by assembling the platform and recording the couch position at each discrete position. The positions were saved to a graphical user interface (GUI) in MATLAB. The GUI was used to predict where the couch should be positioned to ensure the end of the radial arm was positioned at isocenter. Prior to simulation CT, subjects were imaged via US and positions of the transducer recorded with the same setup as during CT acquisition. IMRT treatment plans were developed to a bladder PTV with 1 cm margins.

### Results

The highly conformal IMRT plans show the necessity for strict patient positioning guidelines. The mechanical motion error of the platform was determined to be within the precision of the treatment couch vrt=0.158±1.08 mm, lng=0.205±0.73 mm, lat=0.16±1.09 mm. CT and US images were coregistered in Oncentra. Use of platform for patient positioning is still under evaluation.

### Conclusion

Next generation designs are currently in production. Improvements will include micron movements in the longitudinal direction and radial arm in addition to a water bolus intended to decouple the transducer–patient interface. IMRT plans are highly conformal and lead to greater probability of geographic miss. Our system is an alternative to radiographic methods of image guidance that we will test in a clinical trial of spontaneously occurring TCC in dogs.

**PO‐BPC‐Exhibit Hall‐06**

## Integration of Reduced Order Constrained Optimization for IMRT Planning Into the Eclipse Treatment Planning System

S Bakr,^1,2*^ H Nourzadeh,^1^ S Tuomaala,^3^ L Happersett,^2^ J Yang,^2^ R Radke,^1^ A Jackson^2^

*Rensselaer Polytechnic Institute,^1^ Troy, NY, Memorial Sloan‐Kettering Cancer Center,^2^ New York, NY, Varian Medical Systems,^3^ New York, NY*

### Purpose

We describe the integration of the reduced order constrained optimization (ROCO) algorithm, a previously developed algorithm for accelerating IMRT optimization, into a commercial treatment planning system (Eclipse v13.5). While previous implementations of ROCO were successfully applied to IMRT planning in different sites, these were demonstrated using an in‐house, not commercially available treatment planning system.

### Methods

ROCO suggests a systematic way to characterize the intensity space in a compact form. This enables forming a computationally tractable constrained optimization, which directly enforces hard clinical constraints on organs at risk (OARs) and planning target volumes (PTVs). ROCO starts with sampling of the intensity space by solving a set of unconstrained optimization problems. Principal component analysis (PCA) is used to attain a reduced order representation of fluence profiles, and the dose corresponding to each principal component is computed. Then, a constrained optimization problem is formed and solved over the basis coefficients spanning the reduced size space. The new implementation supports a variety of clinically relevant constraints. It also exploits a previously used iterative scheme to automatically accommodate dose volume constraints. The resulting fluence map is imported back into Eclipse for deliverability assessment, final dose calculation, and plan evaluation. ROCO is developed as a stand alone application in .NET framework 4.5. It uses Eclipse scripting API V13.5 to interact with optimization and dose calculation modules. The new pipeline harnesses asynchronous and multithreaded programming techniques, as well as highly optimized libraries and solvers.

### Results

The ROCO Eclipse implementation is tested on a nonnodal prostate case resulting in a clinically acceptable plan, according to our institute's latest clinical protocol.

### Conclusion

The new integration adds distinctive features that make it more practical in a clinical setup, where the planners can potentially save hours of tedious trial and error to devise clinically relevant IMRT plans.

We acknowledge the collaboration of Varian Medical Systems in carrying out this work. This work was supported in part by Grant Number R01CA148876‐02 from the 401 National Cancer Institute (NCI).

**PO‐BPC‐Exhibit Hall‐07**

## The Wait Is Over for Universal Plan Check Automation

T Halabi,^1*^ H Lu,^1^ D Bernard,^2^ J Chu,^2^ M Kirk,^3^ R Hamilton^4^

*Massachusetts General Hospital,^1^ Boston, MA, Rush University Medical Center,^2^ Chicago, IL, Mass General/North Shore,^3^ Danvers, MA, University Arizona,^4^ Tucson, AZ*

### Purpose

To free thousands of medical physicists from the mechanical labor of plan checks while reducing the likelihood of treatment errors. In doing so, we hope to engage a Moore's law of sorts for automation, as these time savings can be committed towards more automation effort.

### Methods

A novel solution for plan check automation is deployed through the web using ClickOnce technology. With a single click the user downloads, installs, and launches the application. The program uses a patient's Medical Record Number, inputted by the user, to fetch plan PDFs uploaded into MOSAIQ. It then performs two types of checks: 1) comparisons between an arbitrary set of data sources that include the plan PDF and the MOSAIQ database, and 2) more complex checks listed in an automated check box list. It currently works with MOSAIQ, Pinnacle, Eclipse, XiO, proton‐XiO, and RayStation.

### Results

The program is currently in use at 4 facilities. It has checked over 4000 plans. Any facility using MOSAIQ is now a few clicks away from running an automated solution for plan checks. For a typical IMRT plan at Massachusetts General Hospital, the program performs around 600 comparison checks and 100 check box checks. The choice of checks included is informed by the combined clinical physics experience of the program's current 35 users (growing).

### Conclusion

Exponential growth in automation will only occur if solutions are made available to others. By basing the choice of checks included on user suggestions, we've also established a virtuous cycle in which quality is proportional to number of users and vice versa.

**PO‐BPC‐Exhibit Hall‐08**

## Daily Quality Assurance of Coincidence Between Imaging and Radiation Isocenter

Y Huang,^*^ B Zhao, I Chetty, J Gordon, N Wen

*Henry Ford Health System, Detroit, MI*

### Purpose

The targeting accuracy of an image‐guided treatment depends crucially on the coincidence between imaging and radiation isocenter. In this study, we developed and implemented an efficient quality assurance (QA) procedure that measures the coincidence between imaging and radiation isocenter daily.

### Methods

A two‐step procedure was implemented on a Novalis TX. First, four Winston‐Lutz (WL) portal images at gantry angles of 0°, 90°, 180°, and 270° are acquired of a BB that is positioned according to laser or light field close to machine isocenter, the analysis of which provides the offset of the BB relative to the average radiation isocenter (V_1_). Next, the BB was imaged with the ExacTrac X‐ray (V6.0.5, BrainLAB AG) or cone‐beam computed tomography (CBCT) of the OBI system (V1.5, Varian Medical Systems), the analysis of which provides the offset of the BB relative to imaging isocenter (V_2_). The vector, $V_{1}-V_{2}$, is then the deviation between the imaging and radiation isocenter.

### Results

Averaged over a period of a month, the overall deviation to the average radiation isocenter ((μ±σ)) is (0.17±0.10) mm for the ExacTrac X‐ray system and (0.51±0.26) mm for the OBI CBCT on the Novalis TX.

### Conclusion

Both ExacTrac X‐ray and OBI CBCT isocenters on the Novalis TX are reproducible and closely match the average radiation isocenter. The congruency between imaging and radiation isocenters can be efficiently measured as part of daily QA program to ensure accurate patient positioning.

**PO‐BPC‐Exhibit Hall‐09**

## The Effect of Varying Target Size in Eclipse in Dose Calculation Accuracy for Small Fields

Y Qin,^*^ H Zhong, N Wen, A Doemer, J Gordon, I Chetty

*Henry Ford Health System, Detroit, MI*

### Purpose

To investigate the dosimetric impact of varying effective target size in Eclipse on the accuracy of the AAA and AcurosXB algorithms for dose calculation of small fields, using Monte Carlo (MC) simulations and measurements.

### Methods

The effective target size parameter was tuned to 0, 1, 2, and 3 mm in the beam model for AAA v11 and AcurosXB v11 for the 6X flattening filter‐free (FFF) beam of the Edge Linear Accelerator (Varian Medical System, Palo Alto, CA). Dose calculation was performed in a virtual water phantom for 4 fields: 10×10, 2×2, 1×1, and 0.5×0.5 at 100 cm SSD. An IAEA‐compliant phase space file generated based on camera measured spot geometry was set as input into the EGSnrc/BEAMnrc MC code to simulate particle transport in jaws. Subsequently, dose in the phantom was calculated using DOSXYZnrc. Gafchromic EBT3 film measurement was done at 100 cm SSD, 10 cm depth in solid water. Measured and MC doses were compared against calculations with varying target. Percent depth dose and profiles were evaluated.

### Results

Agreement against MC within 2% was observed for all targets at 10×10 and 2×2 fields. Significant differences exist for field sizes less than $2\times 2\;{\text{cm}}^{2}$. At $0.5\times 0.5\;{\text{cm}}^{2}$, dose at $d_{\text{max}}$ differed by 50% between target sizes of 0 and 3 mm for both algorithms; $d_{\text{max}}$ depth differed by 2 mm. MC simulations were validated against film measurements. Varian's recommended spot sizes in Eclipse are 0 mm for AAA and 1 mm for AcurosXB. Our results show that MC simulation agrees most with AAA 1 mm and AcurosXB 1 mm. 3%/1 mm gamma analysis between AAA 1 mm and MC profiles at 10 cm yields 92.6% ($0.5\times 0.5\;{\text{cm}}^{2}$), 90.7% ($1\times 1\;{\text{cm}}^{2}$), and 99.1% ($2\times 2\;{\text{cm}}^{2}$). Between AcurosXB and MC, gamma indices are 77% ($0.5\times 0.5\;{\text{cm}}^{2}$), 86% ($1\times 1\;{\text{cm}}^{2}$), and 83.6% ($2\times 2\;{\text{cm}}^{2}$).

### Conclusion

Dose calculation using AAA and AcurosXB utilizing varying target sizes shows significant difference at field sizes smaller than $2\times 2\;{\text{cm}}^{2}$. The optimal target size is 1 mm for both algorithms based on MC simulations.

**PO‐BPC‐Exhibit Hall‐10**

### Lung Toxicity Versus CT Number Variation by Hypofractionated Lung Stereotactic Body Radiotherapy (SBRT)

K Dou,^1*^ B Li,^1^ M Jacobs,^2^ B Laser,^2^ F Lerma,^1^ M Sarfaraz^1^

*RadAmerica Mercy Radiation Oncology,^1^ MedStar Health, Baltimore, MD, Mercy Medical Center,^2^ Radiation Oncology, Baltimore, MD*

### Purpose

To study lung injury or toxicity from hypofractionated lung stereotactic body radiotherapy (SBRT) by correlating the lung density variation with computed tomography (CT) number following the hypofractionated lung SBRT.

### Methods

Six patients were selected for the retrospective research. Cone‐beam CT (CBCT) was acquired for obtaining the CT number and then getting the lung density. CBCT numbers were corrected using a CIRS density phantom. Each case had a free‐breathing simulation CT followed by a respirationgated 4D CT using a Philips CT Big Bore scanner with a Varian RPM respiratory gating system. The internal target volume (ITV) was created from ten phase‐gated CT images, followed by exporting to the Varian Eclipse TPS for treatment planning with the free‐breath and ten phased images. The planned dose was delivered with a 6 MV flattened filter‐free beam of a Varian TrueBeam accelerator.

### Results

CBCT numbers were sampled from three locations: visual lung tumor (GTV), normal lung in the tumor seated lobe obtaining 30% of the prescribed dose, and reference lung in the normal, tumor‐free lobe. The CT number of the lung tumor was found to significantly increase in a range of 7% to 313% with a radiation scheme of 10 Gy by 5 fractions every other day. However, both normal lungs showed a smaller variation in the CT number than that of the tumor. A maximum increase in CT number of post 5th fraction/prior treatment was 313%, which leads to more than three times increase in lung density after SBRT treatment.

### Conclusion

CBCT can be utilized for not only patient alignment and target localization, but also acquirement of CT numbers of the targeted volume, and the toxicity or injury can, therefore, be associated with the lung CT number variation deduced from CBCT.

**PO‐BPC‐Exhibit Hall‐11**

## A Patient‐Specific Heterogeneous Tumor Model for Glioblastoma Multiforme

C McGuinness,^*^ R Noble

*SLAC National Accelerator Laboratory, Menlo Park, CA*

### Purpose

Gliomas tumors proliferate and invade healthy brain tissue rapidly, yielding short life expectancies. Radiation therapy is commonly used against the disease, but gliomas are known to be radio‐resistant, and almost always recur following treatment. Current radiotherapy protocols are based on the classic linear‐quadratic radiobiological model describing tumor response to radiation, assuming a homogeneous (one‐component) tumor. Gliomas are very heterogeneous, consisting of normoxic, hypoxic, and necrotic tissues, each responding differently to radiation. An enhanced linear‐quadratic model, which takes into account the different responses of the heterogeneous tumor regions to radiation, can guide treatment planning to optimize dose distributions to maximize the therapeutic effect.

### Methods

We used a set of differential equations to model the growth of glioma tumors. Our model expands on the one component model developed by Rockne et al.^(1)^ by including normoxic, hypoxic, and necrotic components and radiosensitivity values for each component separately. Proliferation and diffusion parameters are extracted by contouring the tumor on two sets of pretreatment MRI images and modeling it as a volume equivalent sphere.

### Results

We compared two examples of glioma tumors presented in the study by Rockne with our three component model and find better predictive capabilities for post‐RT tumor volumes using the three component model. For the first case the one‐component model predicts over predicts the effects of radiation, resulting in a tumor diameter of 2.2 cm, while the three‐component model predicts a diameter of 2.6 cm in agreement with measured values. The second case similarly overpredicts the effects of radiation and shows poor agreement with the measured post‐RT tumor volumes.

### Conclusion

The three‐component model accurately matches tumor growth dynamics derived from MRI images for two example cases. Spatial information about hypoxic, necrotic, and normoxic cell densities are derived from the model providing information needed to more intelligently prescribe dose distributions tailored to a specific patient's tumor.

1. Rockne R, Rockhill JK, Mrugala M, et al. Predicting the efficacy of radiotherapy in individual gliblastoma patients in vivo: a mathematical modeling approach. Phys Med Biol. 2010; 85(12):3271.

**PO‐BPC‐Exhibit Hall‐12**

## A Study of Target Volume and Dosimetry Comparision Between 4D Radiation Therapy Plans and 3D Radiation Therapy Plans

C Ma^*^

*Shandong Tumor Hospital*

### Purpose

The purpose of this work was to determine the dosimetric benefit to normal tissues by tracking liver tumor dose in respiratory gating on one phase of four‐dimensional computer tomagraphy(4D CT) images.

### Methods

Liver tumor tracking each phase plans for ten liver cancer patients were compared to the 3D plans with a merged target volume based on 4D CT image in radiation treatment planning system (TPS). The change in normal tissue dose was evaluated in the plan by using the parameters V5, V10, V15, V20, V25, V30, V35, and V40 (volumes receiving 5, 10, 15, 20, 25, 30, 35, and 40 Gy, respectively) in the dose‐volume histogram for the liver; mean dose for the following structures: liver, left kidney, and right kidney; and maximum dose for the following structures: bowel, duodenum, esophagus, stomach, and heart.

### Results

There was significant difference between 4D GTVs (average 115.71 cm^3^) and ITVs (169.86 cm^3^). When the planning objective is 95% volume of PTV covered by the prescription dose, the mean dose for the liver, left kidney, and right kidney has an average decrease 23.13%, 49.51%, and 54.38%, respectively. The maximum dose for bowel, duodenum, esophagus, stomach, and heart has an average decrease 16.77%, 28.07%, 24.28%, 4.89%, and 4.45%, respectively. Compared to 3D RT, radiation volume for the liver V5, V10, V15, V20, V25, V30, V35, and V40 by using the 4D tracking tumor plans has a significant decrease (p≤0.05).

### Conclusion

The 4D tracking tumor method creates plans that permit better sparing of the normal structures than the commonly used ITV method, which delivers the same dosimetric effects to the target. This analysis indicates that 4D tracking tumor radiotherapy allows a data support for reduction in PTV volume and dose reduction in the OARs for liver tumor patients.

**PO‐BPC‐Exhibit Hall‐13**

## Design and Implementation of a National Radiotherapy Incident and Device Reporting System

B Thomadsen,^1,2*^ J Palta,^1,3,4^ T Mackie,^1,2^ F Rath,^1,2^ R Kapoor^3,4^

*Center for the Assessment of Radiological Sciences,^1^ University of Wisconsin,^2^ Madison, WI, Virginia Commonwealth University,^3^ Richmond, VA, VCU Health System,^4^ Richmond, VA*

### Purpose

To design and implement a national incident reporting system for radiotherapy.

### Methods

The design of the reporting system made use of: 1) experience in both industrial and medical event reporting; 2) the dataset taxonomy generated by the AAPM‐led panel; and 3) proven methods to avoid several common problems in such systems: a. Incomplete data due to data‐entry fatigue on the part of the person entering the data; b. Missing data from asking question for which the response requires data that is difficult to obtain or not familiar to most respondents, such as taxonomic classifications; c. Missing reports due to fear of reprisal; d. Selection bias for incidents entered into the larger, national database.

### Results

1. The system is organized so a person from a facility could notify the database agents through a very brief online application, either as an official reporter of an event or anonymously. Anonymous reporters can give contact information with a guarantee of confidentiality.
2. An agent for the database calls the facility and completes the data entry for the facility while discussing the details of the incident. This not only ensures data completion, but also helps the analyst understand the event. 3. Off‐line, the analyst performs a root‐cause analysis and drafts a report with suggested actions to improve quality and safety. 4. The analyst discusses the proposed actions with the facility to assess the practicality in the given setting and together they generate an action plan. 5. While the facility uses its reports for its local database, all the incidents go into the national database, for which the de‐identified data is searchable. 6. The system also includes reporting for equipment problems, and leads to working with vendors to address device issues.

### Conclusion

With the design and implementation of the system, the clients are supported and data managed robustly.

Partial funding for the software development came from TSG Integrations.

**PO‐BPC‐Exhibit Hall‐15**

## Proton Pencil Beam Planning Optimization on Monitor Unit Threshold Setting and Target Coverage in Conformality and Uniformity

D Freund,^*^ J Syh, X Ding, J Syh, B Patel, x song, J Zhang, H Wu, L Rosen

*Willis‐Knighton Medical Center, Shreveport, LA*

### Purpose

To emphasize differences between standard proton pencil beam scanning (PBS) treatment plan versus hybrid plan. The threshold setting of monitor unit (MU) optimizes the plan with efficient spot monitor unit and improve efficacy with less number of energy layer to benefit plan quality.

### Methods

A proton treatment plan needs to meet the proton machine characterization. Various threshold of monitor unit was initiated without sacrificing plan quality. It was also determined that the number of energy layer can be decreased to decline low count spots (i.e., increases of spot MU efficiency in hybrid plan). Specifically, certain energy layers were reoptimized. The little weighting of MU was removed because of scarce spreading of beam spots with low MUs.

### Results

There are three main outcomes of plan quality to be probed. Firstly, the minimal required MU/fraction threshold was inserted. Secondly, spot spacing and spots layer by layer were all countable for the contribution of plan quality. Thirdly, total number of energy layer and the relative weighting of energy layer of each delivered beam need to be explored and evaluated. DVHs of PTV_Evaluation in our cases were compared for each separate plan.

### Conclusion

Spot size is equivalent to the relative dose in the beam delivery system. The energy layer is associated with the depth of beam penetrates. More of beam layer count and fine tiny inefficient spots are not necessary to generate a high‐quality PBS treatment plan. In the opposite, it might degrade the plan quality and might increase the chance of beam pauses during MU delivery. Our work is to maintain the best possible quality plan by beam spot threshold optimization. To keep integrity and harmony among all elements, such as spot size, spot number, layer number, and the relative weighting of each layer in all beams, is important in this study.

**PO‐BPC‐Exhibit Hall‐16**

## QA BeamChecker Plus for the Daily Quality Assurance of a CyberKnife

J Gersh^*^

*Gibbs Cancer Center & Research Institute ‐ Pelham, Greer, SC*

### Purpose

Described is the utilization of the QA BeamChecker Plus (BC+ , Standard Imaging Inc., Middleton, WI), for the efficient and consistent test of the CyberKnife's (CK) output constancy.

### Methods

To use the BC+ with the CK, a water‐equivalent extension is attached to the BC+. This contains four fiducial markers on the same coronal plane, appearing with acceptable separation on the CK's stereoscopic imagers. The user first attaches the CK extension to the BC+ and CT scan the phantom in a technique consistent with the density table setup in the treatment planning system. Following export of the imaging study and import into the MultiPlan TPS, five targets are contoured in the coronal plane (center, superior, left, inferior, and right), each representing the central and peripheral‐most plane‐parallel ionization detectors of the BC+ unit. During planning, single‐path isocentric targets are placed on each of the five detectors. For each of the targets, 500 MUs are delivered. Baselines are acquired and automatically corrected for temperature and pressure.

### Results

Testing uncertainty in repeat measurements, ten measurements were performed immediately after setting baselines. Average output was +0.25% with an SD of 0.11%. On the same day as baseline acquisition, ten acquisitions were delivered with a change in the position of the BC+ system. This required the treatment localization system to recalculate translation and rotation, as well as the positional adjustment of the treatment nodes of the accelerator. By performing this study on the same day as the baseline acquisition, output variation is suppressed. The average output was +0.30% high with an SD of 0.13%. In a three‐week study, average output was +0.23% with an SD of 0.12%.

### Conclusion

When used in conjunction with the CK‐specific extension, the QA BeamChecker Plus provides the user with an efficient and consistent means for the daily verification of beam output.

**PO‐BPC‐Exhibit Hall‐18**

## The Truth About Tobacco: Radiation Cigarettes That Lead to Dose Buildup in Lung Tissue and Cause Cancer

S Enright^*^

*University of Miami, Coral Gables, FL*

### Purpose

According to the CDC, lung cancer is the second most common cancer and is the leading cause of cancer death in the United States. About 90% of lung cancers are attributed to smoking cigarettes. The EPA states that tobacco contains radioactive polonium‐210 and lead‐210 which are alpha emitters. Most of this is from high‐phosphate fertilizers used by the tobacco industry. The purpose of this study is to determine if cigarettes made with tobacco grown with high‐phosphate fertilizers have a significantly higher level of radiation (CMP) when compared to cigarettes made without high‐phosphate fertilizers.

### Methods

Cigarettes purchased locally were divided into two groups: those with tobacco grown with high‐phosphate fertilizers (Group A) and those without tobacco grown with high phosphate fertilizers (Group B). For each sample, 15 cigarettes were placed in a plastic cylinder with two open ends. Filter paper and a vacuum were placed on the bottom of the cylinder and the cigarettes were lit. Once the cigarettes were completely spent, the filter paper was placed on a wipe test plate 1 cm under a Geiger‐Muller counter. Ten‐minute timed counts were taken for each sample.

### Results

The results showed group A had an average of 52.4 CPM and group B had an average of 47.9 CMP. A *t*‐test was performed and the data showed a statistically significant difference in CMP between the two groups for a 95% confidence interval.

### Conclusion

Cigarettes which use tobacco grown with high‐phosphate fertilizers have a significantly higher level of radiation when compared to cigarettes without high‐phosphate fertilizers. Therefore, these cigarettes cause more radiation dose to build up in the lungs of smokers and may play a major role in whether the person develops lung cancer.

**PO‐BPC‐Exhibit Hall‐19**

## Whole Abdominopelvic Treatment of Desmoplastic Small Round Cell Tumor Using Multi‐Isocenter Volumetric‐Modulated Arc Therapy

C Abing,^1*^ P Conway^2^

*Gundersen Lutheran Medical Center,^1^ La Crosse, WI, Gundersen Health System,^2^ La Crosse, WI*

### Purpose

To treat whole abdominopelvic irradiation using a multi‐isocenter volumetric‐modulated arc therapy (VMAT) technique. This technique will be utilized to lower the mean kidney, liver, and pelvic bone dose, respectively. This will demonstrate a dosimetrically superior plan when compared to a conventional anteroposterior technique.

### Methods

A three‐isocenter VMAT plan was created using the Eclipse treatment planning system with the analytic anisotropic dose calculation algorithm. Each isocenter shared the same anteroposterior and lateral position, but were separated by 12.5 cm longitudinally. Each isocenter contained two to three VMAT fields, each with 359.8° of rotation, thus making the entire plan composed of 8 VMAT arcs. The target was the entire abdominopelvic cavity and the organs at risk (OAR) were the bilateral kidneys, liver, and pelvic bones. Because of the unique physical length and complexity of this treatment, special care was taken to ensure the patient was not rotated prior to treatment. This VMAT plan was compared with a conventional treatment plan with two opposed fields, an anterior field with partial transmission block over the liver and a posterior field with partial transmission blocks over the kidneys.

### Results

The three‐isocenter VMAT plan gave lower bilateral mean kidney dose (12.8 Gy vs. 14.3 Gy) and a lower mean pelvic bone dose (15.2 Gy vs. 20 Gy). The liver was optimized to give a mean dose of 24 Gy; this would not have been achievable with the conventional plan. Although treatment time for the VMAT plan was longer than conventional, it was kept to 9 min of “beam‐on” time, with a total treatment time mean and standard deviation of 30 min and 13 min, respectively.

### Conclusion

This methodology proved to be effective and efficient when compared to a conventional field arrangement. Proper care must be taken to ensure accurate isocenter separation and patient rotation.

**PO‐BPC‐Exhibit Hall‐20**

## Variations of Cardiac Dose at Different Respiratory Status in CyberKnife M6 Treatment Plans

S Long,^1*^ C Shang,^1,2^ G Evans,^1^ T Leventouri^1^

*Florida Atlantic University,^1^ Boca Raton, FL, Boca Raton Community Hospital,^2^ Boca Raton, FL*

### Purpose

CyberKnife robotic‐assisted radiation delivery has become a choice for accelerated breast RT, while a slightly increased cardiac dose has been reported. However, the dose dynamics throughout the respiration cycle has scarcely been explored. This study was designed to investigate the dose changes at each respiratory phase or status during respiration circle.

### Methods

Two patients with 4D CT studies and two patients with a pair of free‐breathing and deep breath‐hold CT sets were used for dosimetry comparisons. 4D CT sets were obtained by Siemens CT and its respiratory gating system, comprising of 8 phases. Standard APBI plan at 340 cGy was done per fraction per NSABP B‐39/RTOG 0413 (Vicini and White, 2007) and modulated with CyberKnife M6 on MultiPlan 5.1.2. For the purpose of this study, the tumor volume was outlined in the media‐lower quadrant of the left breast.

### Results

The heart doses are significantly reduced in well inhaled phases, especially for 1–3 cc higher dose regions with 18.7%±4.2% (p<0.01) reduction from averaged doses in other less inhaled phases. Since the dose conformity in lower dose levels are degraded due to its less toxic nature, D5 cc of the heart showed a less sensitive to phase changes during respiratory circle. Compared with free breathing, the deep inhaled breath hold reduces the cardiac dose by 80% and 55%, respectively, in case 1 and case 2.

### Conclusion

In this study although ineligible cardiac doses are noted in APBI plans using 4D free‐breathing CT and instantaneous free‐breathing CT series, the cardiac dose reduction was seen in well inhaled breathing phases and, more significantly in plans with BH CT. This provides practical guidance for cardiac dose reduction applicable with CK M6 APBR.

**PO‐BPC‐Exhibit Hall‐21**

## Compact Proton Team Infrastructure for a Single Room Pencil Beam Scanning Proton Center

X Ding,^*^ J Syh, J Syh, B Patel, X Song, J Zhang, D Freund, I Rosen, H Wu

*Willis‐Knighton Medical Center, Shreveport, LA*

### Purpose

With single‐room proton centers increasing throughout the world, it is very important for the administration and hospital to determine the appropriate size of the proton team to support the state‐of‐art proton unit with PBS technique. Based on our half‐year operation with IBA ProteusONE, the first compact PBS proton unit in the world, we recommend a compact proton infrastructure which could ensure the single‐room proton center's daily clinic operation, commission, and technique development. For the machine acceptance and commission, we would recommend 2 to 4 physicists working two‐shift. The basic acceptance and commission normally take around 2 to 3 months. Currently, we are treating 10 patients per day with 60% prostate patients, 40% cranial and head&neck patients. The infrastructure of the current ProteusONE proton team includes: 1 full‐time physicist for proton planning and daily clinic operation, 1 full‐time physicist for chart checking and QA, 0.5 physicist for continuing technique development, and 2 therapists for daily treatment. Although currently our proton unit uptime is 98.5%, proton machine is a complex unit which has some unexpected down time due to the technique challenges. It is very necessary for the individual clinic to establish contingency plan in case of the proton machine down time. For a hospital to consider building a team to operate a single‐room PBS proton unit for daily operation treating 20–25 patients: 2.5 proton physicists. During daily operation: 1. Oversee the daily proton therapy performance; 2. Planning support physicist ensures the plan accuracy and robustness; 3. Chart Check and patient QA. During machine commission: 1. In charge of CT calibration and device calibration 2. In charge of PBS beam modeling and commission. QA procedure developments. 3. In charge of planning technique development. 1 to 2 proton dosimetrists, 4 proton therapists.

**PO‐BPC‐Exhibit Hall‐22**

## ESAPI: Automatic Extraction and Analysis of Patient and Plan Data Utilizing the Eclipse Scripting API

M Schmidt,^*^ S Hames, Y Yang, P Klenovsky

*Varian Medical Systems, Las Vegas, NV*

### Purpose

To display the ability of leveraging the Eclipse Scripting Application Programming Interface (ESAPI) to gain access to read‐only database information in Eclipse Treatment Planning Systems (Varian Medical Systems, Palo Alto, CA). ESAPI gives the user the ability to get dosimetric and plan‐specific information, as well as statistical information over multiple patient cases for automated data gathering and analysis using a .Net framework.

### Methods

Multiple programs and scripts were created using C# with Visual Studio Express (Microsoft Corporation, Redmond, WA) in order to access patient and plan information in the Eclipse database through the ESAPI. In the current Eclipse implementation, data can be read from the External Beam Planning work space, while future releases include access to other work spaces such as Smart Segmentation, Portal Dosimetry, and MIRS Contouring. Some examples of the programs include the ability to analyze the patient's dose‐volume histogram (DVH) to acquire analytic metrics concerning the patient dosimetry (Dose‐at‐Volume, Volume‐at‐Dose, Homogeneity Index (HI), Conformity Index (CI), etc.), the ability to automate some treatment planning system quality assurance and validation of commissioning data, and the creation of automated patient reports.

### Results

The programs developed could accurately display to the user the exact values from the DVH at given volumes, offering the ability to analyze other dosimetric parameters. Automatic comparisons between measured and calculated data were analyzed, using a gamma comparison, showed promise for TPS commissioning and QA. All of this data is exportable to reports programmatically.

### Conclusion

ESAPI will continue to gain ground as a viable option for exporting data from Eclipse, giving the development community the ability to create personal and creative tools.

The authors are employees of Varian Medical Systems.

**PO‐BPC‐Exhibit Hall‐23**

## Practical Experience Implementing a Linac‐Based Imaging Quality Management Program

J Fagerstrom,^*^ W Culberson

*University of Wisconsin, Madison, WI*

### Purpose

The purpose of this project was to design and implement a comprehensive linac‐based imaging quality management (QM) program for a commercial C‐arm linear accelerator, and to report on the practical experiences involved in commissioning and implementing this part of a radiotherapy center's QM program.

### Methods

A Varian 21EX C‐arm linear accelerator platform including kV and MV on board imaging (OBI) systems (Varian Medical Systems, Palo Alto, CA) was acquired by a university research laboratory. A QM program was designed, based on a literature review of peer‐reviewed journal articles, vendors' user's manuals, and recommended protocols from the AAPM, ACR, and ASTRO, as well as recommendations gathered at Varian Medical Systems' five‐day OBI implementation training course at its training center in Las Vegas, NV.

### Results

Specifics from the literature review and the training course were detailed in a step‐by‐step student manual. This guide included tolerance values consistent with the AAPM TG‐142 recommendations, as well as a single‐page worksheet to record results. The authors intend for this manual (as well as relevant literature review materials) to be made available to interested attendees at the AAPM Spring Clinical Meeting. Five student volunteers reported a QM time burden of 4 hours per involved student. This value is expected to decrease as the team becomes more familiar with equipment, software, and recommended tests.

### Conclusion

Baseline values were collected during the first three months of QM program implementation. Regular monthly images will eventually be evaluated against this initial baseline data, and any failing results will be reviewed by senior lab staff. This project was deemed beneficial to the lab. A QM program was designed and implemented offering students hands‐on experience while achieving and maintaining high standards of quality for the linac‐based imaging systems.

**PO‐BPC‐Exhibit Hall‐24**

## A Dosimetric Validation of a Model‐Based Treatment Planning Algorithm in Lung SBRT

A Meacham,^*^ W Luo, J Molloy

*University of Kentucky, Lexington, KY*

### Purpose

Dose at lung tumor interfaces is not well characterized by model‐based treatment planning algorithms due to electronic disequilibrium. The interface region comprises a relatively large fraction of the total target volume in small lung nodules. This work evaluates the resulting dosimetric uncertainty in stereotactic body radiotherapy of small lung nodules.

### Methods

A Monte Carlo algorithm (MCSIM) based on EGS4 was used to validate dose calculations using the XiO superposition algorithm (CMS). Six lung SBRT plans consisting of 10 or 11 static noncoplanar beams and treated on a Varian 21EX linac were evaluated. GTVs ranged from 0.96 to 10.98 cm^3^ in volume and varied in location within the lung. Planning and treatment parameters were held constant between the XiO and MCSIM plans. Electronic disequilibrium occurring at the lung tumor interface was investigated by contouring a 2 mm ‘peel’ on the outer surface of the GTV. Clinical beam arrangements were studied, as were hypothetical arrangements including small numbers of beams and varied beam energies including 6 and 18 MV. Doses were prescribed to the 80% isodose line. Comparisons of DVHs, mean, minimum, and maximum doses for the GTV, PTV, and corresponding ‘peel’ structures were compared.

### Results

Mean doses in the GTV agreed to within 2.4% and 3.3% for the original 6 and 18 MV plans, respectively. For one patient, the mean GTV dose for a one‐beam plan was within 3%, while the 10‐beam arrangement showed an agreement of 1%. Mean doses in the GTV and 2 mm GTV peel were within 1.5% and 2.5% for both XiO and MCSIM for original 6 and 18 MV plans, respectively.

### Conclusion

XiO superposition performs accurate dose calculation in lung SBRT treatment planning in which a large number of static beams are used. Lower energy (6 MV) is preferable in terms of better agreement to Monte Carlo simulation.

**PO‐BPC‐Exhibit Hall‐25**

## Mobile Shield to Treat Pregnant Patients in Radiation Oncology

V Narayana,^1,2*^ P Wang,^1^ P McLaughlin^1,2^

*Providence Cancer Center,^1^ Southfield, MI, University of Michigan,^2^ Southfield, MI*

### Purpose

To design a fetal shield to treat a pregnant patient's brain using IMRT while protecting the fetus from radiation exposure.

### Methods

A simple mobile shield was designed to encase the fetus in a lead cage. The cage was designed so that the weight of the lead was borne on the floor with no extra weight on the couch. The shield was on wheels and could be easily moved over the patient once the patient lay on the table. Standard lead bricks were stacked on the shield's frame to prevent head and leakage scatter from reaching the fetus. Lead bricks lined the right, left, anterior, and superior of the patient's pelvic area. The treatment plan used for the patient was delivered to a RANDO phantom and the reduction in dose to a point estimated to be the umbilicus of the fetus was estimated using TLD. On the first day of treatment, TLDs were placed at the fundus, umbilicus, and pubis of the patient. Subsequent fractions were delivered with the shield in place.

### Results

TLD measurements were made using a RANDO phantom at 48 cm from the closest jaw edge with and without shielding. RANDO measurements indicated a 56% reduction in dose with shielding. TLD measurements made during treatment with shielding at the fundus, umbilicus, and pubis at 37 cm, 48 cm, 66 cm from the closest jaw edge indicated a dose of 2.7 cGy, 1.4 cGy, and 1.1 cGy, respectively, over the entire course of treatment.

### Conclusion

The head leakage contribution from treatment was adequately reduced to deliver an effective IMRT treatment to the patient while protecting the fetus from radiation exposure.

**Professional Symposium Ballroom D**

***Regulatory Update***

**SA‐A‐BRD‐0**

## Regulatory Update

C Haney,^*^ M O'Hara,^*^ L Fairobent^*^

*AAPM, College Park, MD*

There are many potential changes that could impact the medical physicist and their role in the use of radioactive materials and machines that produce radiation in the practice of medicine. The purpose of this session will be to understanding recent reorganizations at the U.S. Nuclear Regulatory Commission and the Food and Drug Administration as they impact the use of radioactive materials and machines that produce radiation in the practice of medicine.

In addition we explore potential legislative changes for the new Congress and recent state‐based regulatory and legislative changes impacting the practice of medical physics.

**Learning Objectives**:

1. Participants will become familiar with key regulation and guidance changes for the use of radioactive materials in medicine that are under consideration.
2. Participants will provide feedback on proposed changes to presenters.
3. Participants will be prepared to facilitate engagement of their facility and colleagues in the ongoing dialogue about changes.

**Therapy Symposium ‐ SAM Ballroom D**

***Brachytherapy***

**SA‐B‐BRD**

## Brachytherapy

J Schwarz,^1*^ J Esthappan,^1*^ R Cormack^2*^

*Washington University School of Medicine,^1^ St. Louis, MO, Harvard Medical School,^2^ Boston, MA*

For decades, intracavitary brachytherapy (ICBT) treatment planning for cervix cancer has been based on planar X‐ray imaging, which was limited to the definition of dose tracking points relative to radiopaque structures (e.g., the implant and the bony anatomy). Around the 1990s, some clinics transitioned to computed‐tomography (CT) imaging for ICBT, facilitating visualization of the applicator in the uterus and contouring of the organs at risk, but still limited with regard to delineation of the tumor volume itself. More recently, some clinics have begun using magnetic resonance (MR) imaging for ICBT treatment planning, enabling visualization of the tumor. In this session we will review the clinical aspects of MR image‐based ICBT of cervix cancer with regard to target volume definition, dosimetry, and clinical outcomes. Then we will describe the implementation of some of these concepts into a multisequence MR image‐based technique for target definition and adaptive ICBT planning. Finally, this session will discuss the quality assurance (QA) and commissioning requirements for MR image‐based ICBT.

**Learning Objectives**:

1. Understand the clinical aspects of MR image‐based ICBT treatment of cervix cancer
2. Understand how MR imaging can be used for target definition and adaptive ICBT treatment planning
3. Understand the QA and commissioning process for MR image‐based ICBT

J. Schwarz: Defining Targets for Brachytherapy

J. Esthappan: Use of MR for Brachytherapy Target Definition and Planning

R. Cormack: QA and Commissioning of MR for Brachytherapy

**Diagnostic Symposium ‐ SAM Ballroom C**

***Best Practices in Pediatric CT Physics***

**SA‐B‐BRC**

## Best Practices in Pediatric CT Physics

K Strauss,^1*^ R McKinstry^2*^

*Children's Hospital Medical Center,^1^ Cincinnati, OH, Washington University School of Medicine,^2^ St. Louis, MO*

The management of image quality and radiation dose during CT scanning is dependent on how well one manages the radiographic techniques as a function of the type of exam, type of CT scanner, and patient size, from the smallest 2 kg infant to large bariatric patients. The CT scanner's display of expected CTDIvol patient is complete and provides the operator with a powerful tool prior to the patient's helical or axial scan to identify and manage appropriate CT techniques, provided the department has established appropriate diagnostic reference levels (DRLs). This paper provides a step‐by‐step process that allows the development of DRLs as a function of type of exam, of actual patient size, and of the unique radiation output of each CT scanner in a department. Abdomen, pelvis, thorax, and head scans are addressed. Patient sizes from newborns to large adults are discussed. The method addresses every CT scanner regardless of vendor, model, or vintage. Adjustments to techniques to manage the impact of iterative reconstruction are covered. A method to handle all available voltages other than 120 kV is provided. This level of management of CT techniques is necessary to properly manage radiation dose and image quality during CT scans over the wide range of patient sizes that are imaged in the majority of departments on a daily basis.

**Learning Objectives**:

1. Understand the basic steps necessary to develop radiographic techniques for standard adult patients on the variety of CT scanners within a given department
2. Understand the steps necessary to modify radiographic techniques for adult patients for both pediatric and bariatric patients and all sizes in between
3. Learn how to adapt these basic techniques using 120 kV to all available high voltages and to scanners using iterative reconstruction

Keith Strauss serves as a paid consultant for Philips Medical Systems upon request.

K. Strauss: Establishing DRLs in Pediatric CT

R. McKinstry: Pediatric CT Physics: A Radiologist�s Perspective

**Therapy Symposium ‐ SAM Ballroom D**

***Treatment Planning Fundamentals***

**SA‐C‐BRD**

## Treatment Planning Fundamentals

R Howell,^1*^ M Matuszak,^2*^ I Chetty^3*^

*UT MD Anderson Cancer Center,^1^ Houston, TX, University of Michigan,^2^ Ann Arbor, MI, Henry Ford Health System,^3^ Detroit, MI*

While it is important to understand fundamental, standard‐of‐care aspects of treatment planning and delivery, the frequent advances in techniques necessitates an improvement in knowledge about new approaches, and how they should be implemented and utilized in the clinical setting. While, the topic of regional node irradiation (RNI) for breast cancer remains hotly debated in the radiation oncology community, there is a trend toward more frequent inclusion on RNI in both breast and chest wall radiotherapy. This presentation will focus on the fundamental physics and dosimetry principles of such treatments and techniques to minimize dose to heart and lungs. Treatment of lung cancers is confounded by issues related to tumor motion and tissue heterogeneity. In the context of stereotactic body radiotherapy (SBRT) for treatment of early stage lung tumors, where small fields are often used, dose calculation accuracy is of paramount importance. Proper planning margins need to be designed to accurately account for respiratory‐induced tumor motion and residual setup errors from image‐guided treatment.

**Learning Objectives**:

1. To learn the fundamentals of breast treatment planning for cases requiring RNI (supraclavicular and internal mammary nodes) including positioning, isocenter placement, beam arrangements and modulation, techniques to minimize dose to organs at risk (e.g., deep inspiration breath hold), and delivery considerations
2. To learn the trade‐off choices in using either VMAT or IMRT, including target size, anatomical location and organs at risk
3. To review fundamental aspects of simulation, planning, and delivery of radiation for treatment of lung cancers; to review the triumphs and challenges associated with current and new approaches for image‐guided treatment of lung tumors in the face of motion

R. Howell: Basics of Breast Planning

M. Matuszak: IMRT Vs VMAT: Does the Choice Matter?

I. Chetty: Challenges of Accounting for Lung in Planning

**Diagnostic Symposium Ballroom C**

***MR Physics: Current Practice and Future Directions***

**SA‐C‐BRC**

## MR Physics: Current Practice and Future Directions

K Huff,^1*^ R Muthupillai^2*^

*Fusion Physics, LLC,^1^ Apollo Beach, FL, St. Luke's Episcopal Hospital,^2^ Houston, TX*

Essential tools for clinical cardiovascular MRI, Raja Muthupillai. Noninvasive imaging has played a crucial role in the diagnosis of heart disease. Echocardiography is widely used to evaluate left‐ventricular function, and valvular disease. Nuclear scintigraphy is used to assess myocardial perfusion and viability, and recent advances in X‐ray‐computed tomography have made it possible to visualize coronary artery anatomy and coronary artery calcium. In the past decade, as a noninvasive imaging option, cardiovascular MR has grown from a being a mere curiosity to becoming a widely used clinical tool for evaluating cardiovascular disease. Cardiovascular magnetic resonance imaging (CMRI) is now routinely used across multiple centers to study myocardial structure, cardiac function, macro vascular blood flow, myocardial perfusion, and myocardial viability. For someone entering the field of cardiac MR, this rapid pace of development in the field of CMRI might make it difficult to identify a cohesive starting point. In this presentation, key cardiovascular imaging techniques that have found widespread clinical acceptance will be summarized. In particular, essential cardiac and respiratory gating techniques that form the backbone of all cardiovascular imaging methods will be described. It is followed by four sections that discuss: (a) the gradient echo techniques that are used to assess ventricular function, (b) black‐blood turbo spin echo methods used for morphologic assessment of the heart, (c) phase contrast‐based techniques for the assessment of blood flow and valvular function, and (d) CMR methods for the assessment of myocardial ischemia and viability. In each section, we briefly summarize technical considerations relevant to the clinical use of these techniques, followed by practical information for its clinical implementation. In each of those four areas, CMRI is considered either as the benchmark imaging modality against which the diagnostic performance of other imaging modalities are compared, or provides a complementary capability to existing imaging techniques. Description of cutting‐edge CMR imaging techniques that are practiced at few academic centers is excluded and the presentation is focused on describing methods that are widely used and are likely to be available in a clinical setting.

**Learning Objectives**:

1. Understand the utility of cardiac and respiratory gating techniques
2. Understand cardiovascular magnetic resonance imaging techniques
3. Understand clinical applications of cardiovascular magnetic resonance imaging

Given that there have been relatively few changes in the current practice of MRI physics, there will be a brief recap of basic MRI physics, as well as the requirements of the Medical Physicist during an annual evaluation of the typical MRI scanner. These requirements include evaluation of Magnetic Field Homogeneity, ACR phantom scanning, Technologist Quality Control, and MRI Safety, which will be discussed in a lengthier fashion. What continues to cause more strife to the practicing physicist is trouble‐shooting typical MRI artifacts and maintaining a working understanding of the alphabet soup that is the clinically used MRI sequence. These issues will be addressed in conjunction with the proposed changes that are on the horizon by various accrediting bodies.

**Learning Objectives**:

1. Understand the basics of MRI physics
2. Understand the best practices in MRI scanner evaluation
3. Understand troubleshooting methods related to artifacts and other common issues

K. Huff: MR Testing and Quality Control

R. Muthupillai: Trending MR Clinical Application: Cardiovascular MR in Assessing Left Ventricular Function and Myocardial Viability

**Therapy Symposium ‐ SAM Ballroom D**

***A Clinical View of Adaptive Therapy***

**SA‐D‐BRD**

## A Clinical View of Adaptive Therapy

K Brock,^1*^ W Mao,^2*^ R Kashani^3*^

*University of Michigan,^1^ Ann Arbor, MI, UT Southwestern Medical Center,^2^ Dallas, TX, Washington University School of Medicine,^3^ St. Louis, MO*

Adaptive radiation therapy has the potential to ensure the optimal treatment is delivered to the patient by accounting for anatomical and functional changes that occur over the course of treatment. Technically, adaptation has been performed for many years clinically, through the resimulation of patients who have noticeable changes and the design of a new treatment plan. However, the use of deformable image registration, dose summation tools, and advanced plan optimization enables the ability to improve efficiency. The introduction of these tools also has the potential to introduce more uncertainties into the process. This session will discuss the commissioning and QA necessary for the safe use of deformable registration for adaptive radiotherapy, strategies and guidelines to determine when and how to adapt, a practical workflow to enable clinical implementation of adaptive therapy, and a discussion of the costs associated with it. Clinical cases will be used to illustrate these concepts.

**Learning Objectives**:

1. To describe how to commission deformable image registration for clinical use
2. To learn how to implement adaptive therapy in a clinical environment and to understand the cost and resources required
3. To learn the strategies and guidelines to determine when to adapt a treatment

K. Brock: Image Registration and Assessment for Adaptive Therapy

W. Mao: Strategies of How and When to Adapt

R. Kashani: Practical Workflow and the Cost of Adaptive Therapy

**Diagnostic Symposium Ballroom C**

***Communication and New Frontiers***

**SA‐D‐BRC**

## Communication and New Frontiers

R Marsh,^*^ A Kesner^*^

*University of Colorado School of Medicine, Aurora, CO*

During their course of work, medical physicists interact with colleagues who have varied professional backgrounds. Colleagues — be they physicians, technologists, nursing staff, service engineers, or vendors — sometimes have a unique misunderstanding of the physics of diagnostic imaging. Often, these misunderstandings have real consequences in patient and staff safety and/or image quality. In this session, we will discuss some of the more eye‐opening comments that have been made to actual medical physicists. We will also discuss ways to engage colleagues in a productive dialogue about the clinically relevant physics of diagnostic imaging and to incorporate these points into teaching of physician residents and other clinical staff.

**Learning Objectives**:

1. Identify some misconceptions about the physics of fluoroscopy systems and the effect on patient and staff dose
2. Identify some misconceptions about CT and their potential negative consequences on clinical care
3. Learn some ways to improve the understanding of physics among your nonphysicist colleagues without alienating yourself

Patient respiratory motion is now the resolution limiting factor in PET thorax imaging, and must be overcome if we are to utilize present and future high‐resolution technologies. Gating has been proposed as a solution for managing the image degradation caused by motion, and can be supported on most contemporary PET machines using vendor‐supplied hardware. However, respiratory gating is failing to be embraced in routine clinical PET imaging practices. We can speculate that this is because of the added effort required to utilize the respiratory gating hardware, and the uncertain benefit the effort provides. As an alternative to hardware‐based gating systems, there have been developments in building data‐driven motion control strategies. Such methods utilize standard (nongated) acquisitions and extract motion information from fluctuations in the raw acquired signal. The information can be used to generate gated images, optimize the signal, and provide new ways to visualize motion, all while imposing no changes to current clinical image acquisition procedures. This lecture will introduce these strategies and compare them to current commercial motion control options. Furthermore, the concept of a data‐driven motion control framework will be examined as a strategy for future innovation.

**Learning Objectives**:

1. Understand the obstacle respiratory motion poses in PET imaging
2. Understand the challenges in generating and utilizing motion corrected images
3. Understand differences in hardware driven and data‐driven motion control strategies

R. Marsh: Don�t Electrocute Me!: Common Misconceptions in Imaging and Radiation Safety and What To Do About Them

A. Kesner: Deviceless Respiratory Motion Correction in PET Imaging � Exploring the Potential of Novel Data Driven Strategies

**SUNDAY, MARCH 8**

**Therapy Symposium ‐ SAM Ballroom D**

***Using Reported Events to Improve Patient Safety***

**SU‐A‐BRD**

## Using Event Reporting to Improve Patient Safety

E Ford,^1*^ S Richardson,^2*^ S Evans^3*^

*University of Washington,^1^ Seattle, WA, Swedish Medical Center‐Tumor Institute,^2^ Seattle, WA, Yale University,^3^ New Haven, CT*

In 2014, ASTRO and AAPM publicly launched a free national reporting system for incidents in radiation oncology known as the RO‐ILS: Radiation Oncology Incident Learning System. This session will share details on what information is submitted through RO‐ILS, how institutions can use the information locally for improvement, as well as the structure that has been established for reviewing incidents and sharing lessons learned. The session will include a live demonstration of the RO‐ILS portal and case studies drawn from incidents reported to the RO‐ILS system. Strategies will be shared for addressing the barriers that are inevitably encountered in starting and maintaining an incident learning program. Of particular interest are strategies for engaging physicians and leadership, and methods for promoting a culture of safety.

**Learning Objectives**:

1. Understand the value and the structure of the Radiation Oncology Incident Learning System (RO‐ILS) and the steps involved in participation
2. Understand and learn about error investigation from a series of case studies
3. Learn strategies to engage radiation oncologists and other department leaders in reporting and learning from events

**Mammography Symposium ‐ SAM Ballroom C**

***Stereotactic Breast Biopsy / Advances in Breast Imaging***

**SU‐A‐BRC**

## Stereotactic Breast Biopsy / Advances in Breast Imaging

W Geiser,^1*^ A Daus^2*^

*UT MD Anderson Cancer Center,^1^ Houston, TX, Gundersen Lutheran Medical Center,^2^ La Crosse, WI*

No abstract provided.

**Therapy Symposium Ballroom D**

***Hands‐on Session: Fault Tree Analysis Workshop***

**SU‐B‐BRD**

## Hands‐On Session: Fault Tree Analysis Workshop

R Siochi,^1*^ P Dunscombe,^2*^ B Thomadsen^3*^

*West Virginia University,^1^ Morgantown, WV, USA, The University of Calgary,^2^ Calgary, AB, Canada, University of Wisconsin,^3^ Madison, WI, USA*

AAPM Task Group 100's report recommends approaching quality assurance from a broader perspective than traditionally common. Fault tree analysis (FTA) is one of the key methods recommended in the report of TG 100 for understanding failures to help identify mitigation methods. The anatomy of a fault tree will be presented, along with descriptions of the key elements. TG 100 techniques will be examined to explain various concepts in the application of FTA. Then, a methodology for addressing faults and handling FTA will be presented with practical examples. In this session, participants will have the opportunity to try a software tool developed for learning to create and use fault tree analyses with clinically relevant examples. Attendees will also have the opportunity to discuss and create their own fault trees for an example process in radiation oncology.

**Learning Objectives**:

1. Learn the key elements of fault tree analysis techniques
2. Understand how a fault tree analysis is applied to clinical examples
3. Gain experience through the use a software tool to perform a sample fault tree analysis as part of a group

R. Siochi: Overview of Fault Trees

P. Dunscombe: Fault Tree Analysis in Task Group 100

B. Thomadsen: Addressing Faults

**Mammography Symposium ‐ SAM Ballroom C**

***Clinical Topics in Breast Imaging***

**SU‐B‐BRC**

## Clinical Topics in Breast Imaging

L Ochoa,^*^ S Holley^*^

*Washington University, St. Louis, MO*

No abstract provided.

**Professional Symposium ‐ SAM Ballroom D**

***Medical Physics Practice Guidelines and Quality Metrics***

**SU‐C‐BRD**

## Medical Physics Practice Guidelines

K Smith,^1*^ P Halvorsen^2*^

*Johns Hopkins Hospital,^1^ Baltimore, MD, Lahey Clinic,^2^ Burlington, MA*

This presentation will describe the purpose, scope, and process for the development of MPPGs, with an expanded description of the two MPPGs related to appropriate supervision of individuals who are not board‐certified physicists, such as medical physics residents and medical physicist assistants. In addition, a review of current protocols on performance tests of medical linear accelerators and assessment of which tests are essential for safe and effective radiotherapy treatments will be discussed. MPPG #8 will describe the requisite tests to ensure safety of patients and staff, to provide high quality radiation therapy treatments, and to reflect the characteristics of modern technology. FEMA tools are used to rank tests from the current protocols to determine severity of harm if a test is not performed, and occurrence and detectability of the failure of a clinical treatment parameter. The scores are noted in addition to approximate time needed to perform each test, whether a particular test should be performed after machine maintenance, regulatory considerations, and whether proceeding when a certain test fails is warranted if other mechanisms are in place to measure clinical parameters. Accrediting organizations, regulatory agencies, and legislators will be encouraged to reference these MPPGs when defining their respective requirements. MPPGs are intended to provide the medical community with a clear description of the minimum level of medical physics support that the AAPM would consider prudent in clinical practice settings. MPPG #8 includes, but is not limited to, staffing, equipment, machine access, and training. The list is not meant to replace previous protocols, but to provide medical physicists with reasonably achievable and clinically effective performance tests. Qualified medical physicists are responsible for implementing recommended quality assurance protocols and, in some cases, are required to follow the protocols exactly by regulatory agencies. The current protocols are evaluated to ensure that recommended performance tests are necessary and relevant and will make the most efficient use of a clinical medical physicists' time.

**Learning Objectives**:

1. Understand the convergence of external factors that are driving the trend toward minimum practice standards in clinical medical physics
2. Understand the purpose and intended scope of the AAPM's Medical Physics Practice Guidelines
3. Understand the considerations regarding appropriate supervision levels in clinical medical physics
4. Understand the considerations regarding implementation of MPPG #8

K. Smith: MPPG #8 Linac QA

P. Halvorsen: Defining Consistent Minimum Practice Standards in Clinical Medical Physics

**Professional Symposium Ballroom C**

***Quality Metrics and Best Practices***

**SU‐C‐BRC**

## Quality Metrics and Best Practices

E Samei,^1*^ P Dunscombe^2*^

*Duke University Medical Center,^1^ Durham, NC, USA, The University of Calgary,^2^ Calgary, AB, Canada*

The increasing demands on clinical medical physicists make voluntary efforts of the medical physicists' role in improving patient care an increasing priority of clinical practice. Quantitative metrics provide opportunities to highlight and be recognized for improved performance. Methodologies and examples of best practices are illustrated for both the imaging and therapy physics applications. The contributions of imaging physicists to clinical imaging operations is expanding and the evolution of health‐care practice dictates that imaging physicists expand their role to incorporate team‐based models of operational engagement. A paradigm is presented that extends traditional equipment testing to incorporate new technologies based upon quantitative imaging and operational optimization accommodating retrospective evaluations of clinical performance. Qualitative metrics in radiation therapy physics will provide examples that integrate incident learning systems, standardization of practice, and the various certification processes into the safety and quality culture of clinical practice. Specific metrics of quality that include various methodologies, and the associated process and outcomes, are presented.

**Learning Objectives**:

1. Recognize the value of incorporating quantitative metrics in the development of best practices
2. Become familiar with recognized methodologies that contribute the development of effective best practice processes and outcomes
3. Identify opportunities to incorporate quantitative metrics into quality improvement programs in order to expedite safe and efficient patient care

E. Samei: Medical Physics 2.0: A Vision for Effective, Meaningful, and Value‐Based Imaging Physics in the Clinical Environment

P. Dunscombe: Quality in Medical Physics and Beyond

**Therapy Symposium ‐ SAM Ballroom D**

***Collective Efforts to Improve Patient Safety Through Change***

**SU‐D‐BRD**

## Collective Efforts to Improve Patient Safety Through Change

B Curran,^1*^ M Huq,^2*^ P Halvorsen^3*^

*Virginia Commonwealth University Medical Center,^1^ Richmond, VA, University of Pittsburgh Medical Center,^2^ Pittsburgh, PA, Lahey Clinic,^3^ Burlington, MA*

While medical physicists individually work to improve patient safety, there have been a number of collective efforts across various professional societies and industries, spanning more than ten years, which promise to bring further enhancements to patient safety. One such effort is the Integrating Healthcare Enterprise in Radiation Oncology (IHE‐RO), which has been led by members of industry, representatives of AAPM and ASTRO, and other stakeholders, to improve connectivity between radiation oncology systems. IHE‐RO has worked to create software structures to increase patient safety including creating the methodology for a plan veto at the treatment unit. From the AAPM perspective, Task Group 100 has applied tools and techniques used in industry towards analyzing radiation oncology processes and recommended a methodology for assessing risks at various steps in a process and for developing a risk‐based quality management program. Because TG‐100 represents a philosophical change in how we approach quality in radiation oncology, national efforts and resources being assembled to support its adoption into clinical practice will be described. Finally, we present one clinical medical physicist's perspective on how TG‐100 may affect how we evaluate QA processes and adapt our existing practices to improve patient safety.

**Learning Objectives**:

1. Understand different IHE‐RO activities, including plan veto, and how they relate to improving patient safety
2. Learn about the key strategies of AAPM TG‐100 and further resources focused on developing risk‐based quality management programs
3. Learn how the concepts in TG‐100 can be used to analyze current clinical practice

Bruce Curran: Travel funded by ASTRO for participation in IHE‐RO activities. Travel funded by AAPM for participation in DICOM activities.

B. Curran: IHE‐RO Efforts to Improve Interoperability of Systems and Patient Safety

M. Huq: The Rollout of AAPM TG‐100: Getting Engaged in Risk Assessment

P. Halvorse: Reviewing Clinical Processes Through the Lens of TG‐100

**Mammography Symposium ‐ SAM Ballroom C**

***Advances in Breast Imaging: Digital Breast Tomosynthesis***

**SU‐D‐BRC**

## Advances in Breast Imaging: Digital Breast Tomosynthesis

J Sabol,^1*^ M Glaser^2*^

*G.E. Healthcare,^1^ Waukesha, WI, Alliance Medical Physics LLC,^2^ Alpharetta, GA*

No abstract provided.

**MONDAY, MARCH 9**

**Therapy Symposium ‐ SAM Ballroom D**

***Improving Linac QA with TG142 and Beyond***

**MO‐A‐BRD**

## Improving Linac QA with TG‐142 and Beyond

E Klein,^1*^ J O'Daniel,^2*^ B Heintz^3*^

*Washington University,^1^ Saint Louis, MO, Duke University Medical Center,^2^ Durham, NC, Texas Oncology,^3^ PA, Southlake, TX*

Six years after publication, full clinical implementation of the AAPM TG‐142 report on accelerator quality assurance remains challenging. First, the choice of methodologies and vendor tools to satisfy TG‐142 requirements is critical to successful implementation. There are many options for hardware and software programs that may or may not be ideal for a particular clinic's needs. Second, there are many QA tests not covered in TG‐142 (i.e., arc therapy techniques) that need the physicist's attention. Third, to achieve a clinic‐specific QA process that is both effective and efficient, it is critical to determine the relative importance of each test. Failure mode and effect analysis (in the vein of TG‐100) allows for a quantitative comparison of each test's clinical impact.

**Learning Objectives**:

1. To learn about the current vendor tools available for TG‐142 testing and to understand the pros and cons of each tool
2. To understand what is still missing from TG‐142 and how to account for these tests in clinical practice
3. To quantify the clinical impact and relative importance of QA tests in TG‐142 via FMEA analysis

E. Klein: What Is Missing in Current TG‐142 Guidance?

J. O�Daniel: Failure Mode and Effects Analysis of TG‐142

B. Heintz: Vendor Tools and Uses for TG‐142

**Diagnostic Symposium ‐ SAM Ballroom C**

***Radiation Dose Metric Monitoring***

**MO‐A‐BRC**

## Radiation Dose Metric Monitoring

R Dickinson,^1*^ M Supanich^2*^

*Texas Health Presbyterian Hospital Dallas,^1^ Dallas, TX, Rush University Medical Center,^2^ Chicago, IL*

In February 2014, the AAPM commissioned a Task Group to “create a practice guideline on the minimum requirements of patient dose tracking systems based on core patient safety objectives.” A diverse group of clinical medical physicists, both diagnostic radiological and medical nuclear, was assembled to create this shopping list of sorts for AAPM and other imaging professionals to utilize and reference when considering the purchase and installation of such a Radiation Dose Metric Monitoring (RDMM) product. This session will review material being published in the Medical Physics Practice Guideline (MPPG), with samples of how the software products may be used in a comprehensive QA program.

**Learning Objectives**:

1. Understand the scope of the RDMM MPPG
2. Have knowledge of common RDMM elements across all imaging modalities
3. Have knowledge of modality‐specific RDMM elements
4. Be exposed to limitations of various RDMs and the MPPG
5. Be presented with ideas for incorporating and leveraging RDMM systems in a QA program

R. Dickinson: MPPG on Radiation Dose Metric Monitoring Systems & How to Use Them�Part 1

M. Supanich: MPPG on Radiation Dose Metric Monitoring Systems & How to Use Them�Part 2

**Young Investigator Symposium Ballroom D**

***Young Investigator Clinical Symposium***

**MO‐B‐BRD‐01**

## Commissioning of a Novel Commercial Motion System to Investigate Dosimetric Consequences Due to Variability of Respiratory Waveforms

A Cetnar,^*^ J James, B Wang

*University of Louisville, Louisville, KY*

### Purpose

The purpose of our study was to assess the feasibility of HexaMotion (ScandiDos, Uppsala, Sweden) for clinical use. The motion phantom with six degrees of freedom was used to evaluate the dosimetric consequences of respiratory waveform variation.

### Methods

The positional accuracy of the HexaMotion was assessed using an independent optical‐guided system and digital level. Dosimetric consequences of waveform variation were investigated using an internal target volume (ITV) approach for planning. A waveform of known amplitude was used by HexaMotion to simulate respiratory motion during a 4D CT. A cylindrical ITV was created to cover the inner diode region of the Delta4 through all 10 phases. The plan was delivered using both the waveform from simulation and a waveform of greater amplitude. The delivery was repeated using gating limits that would terminate the beam if the amplitude of the treatment waveform became greater than the amplitude of the expected waveform from simulation.

### Results

The maximum deviation for translation was 0.3 mm and rotation was 0.5°. The device was able to reproduce a patient waveform with a standard deviation of 0.4 mm. As the amplitude of the waveform increases for treatment delivery, the diodes on the periphery of the target volume receive less than the planned dose. However, by using limits to terminate the beam outside of the original amplitude, the measured dose was similar to the planned dose. The average difference in the ITV region between the planned motion and the larger amplitude was 9.1% less with no gating limits, but only differed 0.1% with the gating limits in place.

### Conclusion

When using the ITV technique for SBRT planning, we recommend the use of gating limits that coincide with the amplitude of the patient waveform at simulation to prevent the potential underdosing of the ITV due to changes in patient respiration pattern.

**MO‐B‐BRD‐02**

## Use of Electronic Portal Imaging Device (EPID) for Quality Assurance (QA) of Electron Beams on Varian TrueBeam System

B Cai,^*^ S Yaddanapudi, B Sun, H Li, C Noel, S Mutic, S Goddu

*Washington University in St. Louis, St. Louis, MO*

### Purpose

EPID has not been utilized to QA electron beam parameters on Varian TrueBeam LINAC due to unavailable imager calibrations and disabled dosimetry imaging acquisition mode for electron beams. This study aims to provide solutions to implement EPID‐based QA for electron beams on Varian TrueBeam system.

### Methods

Ad‐hoc mode electron beam images were acquired in developer mode with XML code. Large source‐to‐imager distance flood field (LSID‐FF) was proposed and used for calibration. Over a two‐month period, images were taken with a proposed diagonal‐wedge phantom for all electron energies using 20 by 20 cm^2^ applicator. Beam parameters including output, field size, symmetry, flatness, uniformity, and percent depth of ionization (PDI) curve were analyzed and compared with baseline images for constancy check. To test the sensitivity of EPID in catching energy change, bending magnet current (BMI) was detuned to allow energy change corresponding to ±1.5 mm change in R50 of PDD. EPID images were then acquired using same method and compared against baseline on multiple machines.

### Results

LSID‐FF calibration method is appropriate for constancy check. After correction, beam profile extracted from EPID is comparable to Sun Nuclear IC‐profiler results. The two‐month EPID data illustrated same trend with SunNuclear Daily QA device. 0.3 changes in uniformity and 2‐unit shift in PDI curve were observed when energy changes exceed TG‐142 tolerance.

### Conclusion

The experimental results clearly demonstrated that electron beam parameters can be reliably measured with EPID for constancy check with proposed imaging acquisition and calibration method and phantom. Using uniformity and PDI shift as metrics, EPID measurement is sensitive enough to catch energy change according to TG‐142 guideline.

**MO‐B‐BRD‐03**

## Feasibility of Using Virtual Unenhanced Images to Replace True Unenhanced Images in Multiphase Renal CT Exams

D Popnoe,^*^ S Zhou, C Ng, E Loyer, H Kang, H Kaur, A Jones

*MD Anderson Cancer Center, Houston, TX*

### Purpose

To assess the feasibility of replacing true unenhanced (TUE) images with virtual unenhanced (VUE) images derived from dual‐energy computed tomography (DECT) data in multiphase renal CT exams, which could allow for reduced patient dose and increased patient throughput.

### Methods

Virtual unenhanced scans were constructed from DECT data using a multimaterial decomposition algorithm for 60 consecutively selected patient studies for a retrospective study. Image quality was assessed for VUE and TUE images qualitatively and the differences were tested with a mixed effects model. CT numbers were measured in the liver, spleen, spine, aorta, cystic lesions, subcutaneous fat, renal cortex, and medulla and the differences between TUE and VUE measurements were tested with a Student's paired *t*‐test.

### Results

Mean CT numbers (±SD) on TUE and VUE images, respectively, were 51.16±10.74 and 52.27±10.16 (p<0.001) for liver, 41.35±6.35 and 47.62±6.13 (p<0.001) for spleen, 155.32±61.91 and 82.15±43.72 (p=1.00) for spine, 35.91±5.7 and 44.76±9.44 (p=0.543) for aorta, 15.97±10.11 and 15.14±10.93 (p<0.001) for cystic lesions, −99.15±15.96 and ‐−95.64±15.31 (p<0.001) for subcutaneous fat, 27.45±5.97 and 40.42±7.41 (p=0.999) for renal cortex, and 29.11±4.79 and 28.1±7.63 (p<0.001) for renal medulla. VUE image quality was noninferior to TUE images in all cases, except in the visualization of major vessels and the depiction of the liver parenchyma.

### Conclusion

This work indicates that, although there is potential for VUE images to replace TUE images, there is still work to be done to identify the optimal set of image acquisition and processing parameters.

CABIR/GE In‐kind research contribution study plan

**MO‐B‐BRD‐04**

## Quantifying Organ Dose and Improving Image Quality in CT Exams of Subjects with Metal Implants Utilizing a Metal‐Artifact Reduction Algorithm

I Lipnharski,^*^ C Carranza, A Mench, R Lamoureux, B Cormack, S Bidari, L Rill, M Arreola

*University of Florida, Gainesville, FL*

### Purpose

To evaluate the effect of metal implants on organ doses and image quality in CT examinations utilizing postmortem subjects.

### Methods

Three similar sized adult female postmortem subjects were scanned on a commercial 320‐slice scanner using a chest‐abdomen‐pelvis protocol. One subject presented metal implants, specifically in the sternum, lumbar, and pelvic regions. All subjects had optically stimulated luminescent dosimeters inserted into various organs, and were scanned using a helical 0.5 mm×80 detector setting, reconstructed with an iterative algorithm (AIDR). CTDIvol, DLP, and organ doses were recorded for each scan. The subject containing implants was additionally scanned using a volumetric 0.5 mm×320 detector setting, reconstructed with AIDR and an artifact reduction algorithm (SEMAR). Image noise was measured for each image series, and image quality was assessed by means of a blind observer study by experienced radiologists.

### Results

The scans of the two implant‐free subjects demonstrated similar doses. Their reported CTDIvols were 6.6 mGy and 6.8 mGy, with a maximum dose difference of 1.9 mGy in the stomach. The scans on the subject containing implants resulted in a higher CTDIvol of 9.7 mGy, and increased dose differences of 3.0 mGy in the stomach, 3.2 mGy in the small intestine, 2.5 mGy in the colon, and 2.3 mGy in the ovaries, relative to an implant‐free subjects. All images were graded to be of diagnostic quality, with the exception of the pelvic region of the subject with hip implants, exhibiting streaking artifacts. Image quality was improved significantly without the need for dose increases when SEMAR reconstruction was employed, improving visualization of the soft tissue and bone structures surrounding metallic implants.

### Conclusion

Patients containing metallic implants may locally experience increased organ doses and impaired image quality. Image quality and diagnostic capabilities may be improved by utilizing metal artifact reduction reconstruction algorithms.

**MO‐B‐BRD‐05**

## Will CyberKnife M6 Multileaf Collimator Offer Advantages Over IRIS Collimator in Prostate SBRT?

V Kathriarachchi,^1*^ C Shang,^1,2^ G Evans,^1^ T Leventouri,^1^ G Kalantzis^1^

*Florida Atlantic University,^1^ Boca Raton, FL, Lynn Cancer Institute,^2^ Boca Raton Regional Hospital, Boca Raton, FL*

### Purpose

CyberKnife M6 InCise multileaf collimator (MLC) has become a new modality in practice. Its ability of forming irregularly shaped beamlets offers a potential for more efficient dose optimization and treatment delivery in comparison with that by IRIS dodecagon beams. This study is focused on quantification of such timesaving ability in prostate SBRT with comparable dosimetry plans.

### Methods

Eight prostate cancer patients were planned in MultiPlan 5.1.2 respectively utilizing IRIS and MLC for 36.25 Gy in 5 fractions. PTV was outlined for treating prostate only. All plans were evaluated by dose conformity index (CI), homogeneity index (HI), new conformity index (nCI), and PTV coverage. In addition, maximum doses at the bladder and rectum, calculated treatment time per fraction, and planned MUs were also compared and tested for significance with the Wilcoxon test.

### Results

In both IRIS and MLC plan groups, PTV Dmax was scaled to 115% while the HI was maintained at 1.15. The mean $V_{100}$ was 95.42% for IRIS, and 95.36% for MLC (p=0.48); mean CI: 1.08 vs. 1.05 (p=0.09); and mean nCI: 1.13 vs. 1.11 (p=0.11). Between the groups, the differences of Dmax for the bladder and rectum were found insignificant (p=0.4). Changing from IRIS to MLC, the average treatment time per fraction was reduced by 35% (43.5±2.6 min vs. 28.3±1.6 min, p<0.01) and the planned MU's were decreased by 40% (50318±8976 vs. 30286±2211, p<0.01).

### Conclusion

This investigation demonstrated the ability of CyberKnife M6 to produce prostate SBRT plans equivalent to those using IRIS in terms of target coverage, and dose sparing of critical structures. However, a significant 35% reduction in treatment time and 40% reduction in number of MUs were achieved by replacing IRIS with MLC without dosimetric compromise in planning quality.

**MO‐B‐BRD‐06**

## Characterization of the Effect of MRI on Gafchromic Film Dosimetry

M Reyhan,^*^ T Chen, M Zhang

*Rutgers University, New Brunswick, NJ*

### Purpose

To characterize the perturbation caused by magnetic resonance (MR) imaging on Gafchromic film used as a radiation detector and develop a correction method.

### Methods

Three sets of Gafchromic EBT2 film were compared: radiation (RAD), radiation followed by MR imaging (RAD+B), and MR imaging followed by radiation (B+RAD). The T1w and T2w MR imaging was performed using a 1.5T scanner with the films in a chicken leg phantom. Doses from 0 to 800 cGy were delivered to each set by a 6 MV Linac. The time interval between radiation and MR imaging was less than 10 min. Film calibration was generated from the red channel from the RAD for control. Microscopic imaging was performed on two pieces of film. The effect of specific absorption rate (SAR) was determined by exposing another three sets of films to low, medium, and high SAR through a series of pulse sequences.

### Results

No discernible preferential alignment was detected on the microscopic images of the irradiated film exposed to MRI. No imaging artifacts were introduced by Gafchormic film on any MR images. On average, 4% dose difference was observed between B+RAD or RAD+B, and RAD with the same calibration curve. The pixel value between the B+RAD or RAD+B and RAD films were found to follow a linear relationship pixel(RAD)=1.02×pixel(B+RAD or RAD+B). By applying this correction, the average dose error was reduced to 2%. The SAR experiment revealed a dose overestimation with increasing SAR even when the correction was applied.

### Conclusion

MR imaging introduces perturbation on Gafchromic film dose measurements by 4% on average, if the film was calibrated without the presence of MRI. This perturbation can be corrected by applying a linear correction to the pixel values. The imaging protocol should be designed to avoid delivering high SAR, for *in vivo* Gafchromic film MR irradiation measurements.

**MO‐B‐BRD‐07**

## Combining Collimation with Spot‐Scanning Proton Therapy to Improve Brain Treatments

E Gelover,^1*^ A Moignier,^1^ B Smith,^1^ D Wang,^1^ R Flynn,^1^ L Lin,^2^ M Kirk,^2^ T Solberg,^2^ D Hyer^1^

*University of Iowa,^1^ Iowa City, IA, University of Pennsylvania,^2^ Philadelphia, PA*

### Purpose

This work investigates the benefits of using a dynamic collimation system (DCS) for penumbra reduction during the treatment of brain tumors with proton spot scanning. The DCS consist of two pairs of orthogonal trimmer blades driven by linear motors; by intercepting the proton pencil beam near the lateral boundary of the target in the beam's eye view, the DCS yields beam spots with a reduced size. To quantify the effect of these smaller spots near the boundary of the target, a comparison of uncollimated and collimated treatment plans has been performed.

### Methods

Spot‐scanning treatment plans for five brain patients previously treated with proton therapy were created in RDX, an in‐house treatment planning system that utilizes the Hong pencil beam algorithm. To insure an accurate starting point for the comparison, the characteristics of the IBA Universal Nozzle were modeled in RDX and used to reproduce the clinical uncollimated treatment plans. Collimated treatment plans were then created by taking into account the effects of the DCS on each beam spot. The plans were reoptimized with the goal of maintaining target coverage and improving conformity. All other parameters (e.g., spot locations, energy layers, air gap) remained unchanged.

### Results

The collimated plans demonstrated almost identical PTV coverage, but a significant reduction in the mean dose to healthy tissue surrounding the target. The average reduction of the mean dose to a 10 mm ring surrounding PTV is 13.77% (95% confidence interval: 11.65%–15.89%, *p*≤0.0001). The conformity index for the 100% isodose line of the collimated plans yielded an improvement of 7.6% in comparison with the uncollimated plans.

### Conclusion

The improvement in lateral penumbra obtained from the use of the DCS in combination with spot‐scanning proton therapy can reduce the dose to healthy tissue surrounding the target while preserving target coverage.

This work is supported by IBA (Ion Beam Applications S.A.)

**MO‐B‐BRD‐08**

## Effect of Small Field Output Factor Measurement Uncertainty on Patient‐Specific IMRT QA as a Function of Plan Complexity

E Laugeman,^*^ J Evans, D Fiedler, S Kim

*Virginia Commonwealth University Medical Center, Richmond, VA*

### Purpose

To evaluate the effect of small field output factor measurement errors on IMRT plan dosimetry and to determine if patient‐specific IMRT QA is able to catch these errors.

### Methods

Output factors (OF) were measured for our 6 MV Varian Trilogy linac with a 0.6cc Farmer ion chamber oriented perpendicular to the beam to mimic a worst case OF measurement scenario. Due to volume averaging and nonequilibrium conditions, this curve differs from our clinical OF curve measured with a CC01 ion chamber for field sizes below $4\times 4\;{\text{cm}}^{2}$. Both OF curves were utilized in Pinnacle Treatment Planning System to recalculate patient‐specific IMRT QA planar phantom dose (IBA MatriXX MultiCube) for a total of 15 clinical IMRT patient plans in four sites: brain, spinal cord, head and neck, and lung. The absolute difference between the two dose maps were calculated using OmniPro I'mRT software. Additionally, each plan was reoptimized with the goal of increasing plan complexity to increase the role of small field OF errors (i.e., increased number of segments and decreased segment size) and the OF phantom dose comparison repeated.

### Results

The two OF curves differed for the 1, 2, 3 cm square field sizes, by 50%, 15%, 1.5%, respectively. Differences in plan dose were much smaller for both the initial clinical patient plans and the increased complexity plans; more than 98% of the pixels had a dose difference of less than 1%. The pixels with differences >1% appeared mainly on the high‐gradient dose regions.

### Conclusion

The error in calculated dose for a Varian Trilogy linac calculated with Pinnacle TPS when using incorrectly measured small field OF values is very small. Due to the poor spatial resolution of the detector and the use of 3%/3 mm gamma passing criteria, this error is not significant enough to be detected with routine patient‐specific IMRT QA.

**MO‐B‐BRD‐09**

## CT Breast Dose Dependence on Breast Volume, Tissue Composition, and Body Circumference: a Monte Carlo Simulation Study

J Holmes,^*^ D Carver, R Price, D Pickens, M Stabin

*Vanderbilt University Medical Center, Nashville, TN*

### Purpose

To determine the dependency of CT breast dose with respect to patient size, patient tissue composition, breast composition, and total breast volume.

### Methods

Computerized human phantoms were generated to simulate female patients of varying size and tissue composition. A Monte Carlo simulation of a 64 slice CT scanner was created using a radiation particle transport software toolkit with CT technique variables kept constant at 120kVp, 100mAs, body bow‐tie filter, helical scan pitch of 1.0, and beam collimation of 64×0.625. Anatomical landmarks for start and stop of a routine chest CT protocol scan kept constant for all phantoms. To simulate varying body size, each phantom was scaled in circumference in the axial dimension only. Breast tissue was simulated as homogeneous mixtures of adipose and glandular tissues. Patient body composition was simulated with varying degrees of excess adipose and soft tissue.

### Results

A linear dependence of breast dose on the percent of glandular tissues was found in simulations ranging from 0% glandular (100% adipose) to 100% glandular. An inverse relationship was found between breast dose and body circumference. As the breast tissue gets closer to the edge of the gantry and farther away from isocenter, the dose to the breast tissue decreases. Breast dose was found to be relatively higher in phantoms comprised of higher fractions of body adipose tissue.

### Conclusion

These simulations demonstrate decreasing breast dose with increasing body circumference. Breast dose increased with increasing percentages of glandular breast tissues and, in large patients, with excess adipose tissue. The latter **Result** is partially explained by the diminished attenuation of the excess adipose tissues throughout a patient's scan. This information could be used to optimize CT scan protocols of the chest region to allow dose savings in young and adolescent women, where breast radiosensitivity is higher.

**MO‐B‐BRD‐10**

## A Model of Tumor Control Probability Based on Tumor Shape and Spatial Distribution of Oxygen

D Goldbaum,^*^ R Hamilton

*University Arizona, Tucson, AZ*

### Purpose

The purpose of our investigation is to improve our qualitative understanding of the relationship between tumor control probability (TCP) and tumor volume (V), and to present a model that will predict TCP using measurements from the patient to be treated.

### Methods

We present qualitative models with phenomenological parameters that can be set by comparison to past patient data. Each model uses a bimodal expression for the cell‐survival fraction, where each clonogen is considered to be either normoxic or hypoxic, and where the radiosensitivity of the hypoxic clonogens is characterized by an average hypoxia reduction factor. In each model we provide an argument for how the hypoxia reduction factor should scale with respect to V. Also, we point out that, in a clinical setting, one can often characterize tumor shape by measuring the tumor's diameter along each of its “principal axes”. We use these parameters to analyze the tumor as if it were an ellipsoid. Then for more in‐depth algebraic analysis, we use the simplifying assumption that the tumor is spherical.

### Results

We generated a model where the scaling relationship between TCP and V is similar to those found by fitting clinical data. This suggests that one can qualitatively understand this scaling through the effect of hypoxia, and that one might be able to use tumor measurements from a single patient to predict the TCP vs. dose relationship for that patient.

### Conclusion

We generated a phenomenological model, based on reasonable qualitative principles, where the scaling of TCP with respect to V resembles that observed in clinical data. We will use TCP vs. V data to determine values for the model's phenomenological parameters. Then we will have a sound basis on which to evaluate its clinical value. We will also analyze the model with respect to tumor hypoxia data.

**MO‐B‐BRD‐11**

## The Feasibility of Using a Knowledge Base of Prior Treatment Plans in Cervical Cancer: a Dosimetric Comparison with Original Plans

C Ma^*^

*Shandong Tumor Hospital*

### Purpose

To demonstrate the feasibility of using a knowledge base of prior treatment plans to generate new cervical intensity‐modulated radiation therapy (IMRT) plans. Dosimetric differences were investigated between knowledge‐based IMRT rapidplans and original plans.

### Methods

A database of 20 cervical IMRT treatment plans was assembled to create a knowledge‐based IMRT rapidplan model. Another 19 clinical cases were randomly selected to test the model. A comparison of the difference in the dose‐volume histograms (DVH) between the semiautomated treatment plans and the original treatment plans were analyzed.

### Results

On average, the new knowledge‐based rapidplans are capable of achieving very comparable planning target volume coverage as the original plan, to within 1% as evaluated for D98, D95, and D1. For the rectum, the mean and standard deviation of the dose percentage differences for D20, D30, and D50 are 3.79%±8.31%, 4.00%±9.87%, and 1.52%±10.89%, respectively. For the bladder, the mean and standard deviation of the dose percentage differences for D20, D30, and D50 are −2.43%±9.40%, −2.03%±10.17%, and −2.94%±12.30%, respectively. For the femoral heads, the mean and standard deviation of the dose percentage differences for the left and right are 3.15%±18.29% and −3.18%±13.79%. A negative percentage difference indicates that the new plan has greater dose sparing as compared to the original plan.

### Conclusion

We demonstrate a knowledge‐based IMRT model in cervical cancer can generate clinically acceptable treatment plans of high quality. This semiautomated approach can improve the efficiency of the treatment planning process, while ensuring that high‐quality plans are developed.

**MO‐B‐BRD‐12**

## Empirical Beam Angle Optimization for Intensity‐Modulated Radiation Therapy in Lung Patients

S Pella,^1^ B Doozan^2*^

*South Florida Radiation Oncology,^1^ Boca Raton, FL, Florida Atlantic University,^2^ Boca Raton, FL*

### Purpose

Empirical methods of beam angle optimization (BAO) are tested against the BAO that is currently employed in Eclipse treatment planning software. Creating a better BAO can decrease the amount of time a dosimetrist spends on developing a treatment plan, improve the treatment quality, and enhance the tools an inexperienced dosimetrist can use to develop planning techniques.

### Methods

The methods used currently to create the treatment plans involve limited use of the BAO that is available, due to the poor results that have been shown. Using empirical data created by experienced dosimetrists from 69 patients in lung cancer treatment, the most frequently used gantry angles were applied to four different regions in each lung to gather an optimal set of fields that could be used to treat future lung cancer patients. This method, given the moniker FAU BAO is compared in 7 plans created with Eclipse BAO choosing to use 5 of a possible 9 beams, as well as forcing the Eclipse optimization to create a minimum of 9 fields.

### Results

The results show that the conformality index improved by 30%, the conformation number improved by 12%, and the organs at risk (OAR) were overall protected to produce fewer nonstochastic effects from the radiation treatment with FAU BAO over the Eclipse BAO.

### Conclusion

A beam optimization method that uses the empirical method is superior to the one provided by the planning system. The planning time is reduced by 35% and the dosimetric results are significantly better. Using this system, the dosimetrists can see an improvement in their plan quality and the time they spend in creating it.

**Professional Symposium Ballroom D**

***Preparing for the MOC Written Exam and Match Program Update***

**MO‐C‐BRD**

## Preparing for the MOC Written Exam and Match Program Update

R Siochi,^1*^ J Rong,^2*^ M Yester,^3*^ J Gibbons^4*^

*West Virginia University,^1^ Morgantown, WV, UT MD Anderson Cancer Center,^2^ Houston, TX, UAB Medical Center,^3^ Birmingham, AL, Mary Bird Perkins Cancer Center,^4^ Baton Rouge, LA*

The ABR MOC written exam has been offered for medical physics for approximately 5 years. About 30% of the material on the exam is core medical physics, technology, and safety in the subspecialty area of medical physics. The other 70% comes from recent advances in the field.

The nature of the ABR MOC exam will be discussed and the statistics from recent exams will be presented. Recent exam takers will present their preparation strategies to help others prepare for the exam.

**Learning Objectives**:

1. Understand results from recent MOC written exams
2. Receive preparation advice for the therapy medical physics MOC exam
3. Receive preparation advice for the diagnostic medical physics MOC exam

In 2014, the AAPM and SDAMPP announced the creation of a medical physics residency‐match program for graduate students and postgraduate trainees. The match began in the fall of 2014 for the residency year beginning 07/01/15.

**Learning Objectives**:

1. Review the history of the MedPhys match program
2. Review requirements for participation in the MedPhys match program
3. Understand preliminary MedPhys match program results

R. Siochi: MOC Exam Preparation ‐ Therapy

J. Rong: MOC Exam Preparation ‐ Diagnostic

J. Gibbons: Match Program Update

M. Yester: MOC Exam Update From the ABR

**Professional Symposium Ballroom C**

***Leadership and Management***

**MO‐C‐BRC**

## Leadership & Management

M Howard,^1*^ R Miller,^2*^ M Meineke^3*^

*Parkridge Medical Center,^1^ Chattanooga, TN, Northwest Medical Physics Center,^2^ Seattle, WA, Ohio State University,^3^ Columbus, OH*

The Leadership and Management session will provide an opportunity for clinical medical physicists to expand their skill sets to encompass those nontechnical aspects of professional growth associated with leadership and management. The session provides discussions of the challenging leadership and administrative roles in which clinical physicists may participate, and a demonstrative presentation of initiating and carrying through conversations with co‐workers, administration, and supervisees. The relationship of management methods, as described by several well‐known leadership programs, will be presented and related to the role of the medical physicist and the associated impacts on clinical care and departmental operations. Extensions to project management and personnel interactions are illustrated by interactive examples that provide examples of constraints associated with staff, budgetary issues, and operational dynamics. The dynamics of personal interactions are illustrated through presentations invoking audience participation and feedback to reinforce examples of effective and ineffective communication methods.

**Learning Objectives**:

1. Develop an appreciation for the evolving leadership and management roles the medical physicists encounters in clinical practice
2. Identify options for optimizing the complex process of project management and its effective implementation to deliver quality patient care
3. Develop interactive skills and strategies that can be employed to successfully address sensitive issues and discussions within the clinical environment

M. Howard: Medical Physics and the Evolving Administrative Role

R. Miller: Can We Talk? Navigating the Minefields of Difficult Conversations

M. Meineke: Can We Talk? Navigating the Minefields of Difficult Conversations

**Therapy Symposium ‐ SAM Ballroom D**

***Detectors for Radiotherapy Measurements***

**MO‐D‐BRD**

## Detectors for Radiotherapy Measurements

S Kry,^1*^ M Chan,^2*^ L Archambault^3*^

*MD Anderson Cancer Ctr.,^1^ Houston, TX, USA, Memorial Sloan‐Kettering Cancer Center,^2^ Basking Ridge, NJ, USA, CHUQ Pavillon Hotel‐Dieu de Quebec,^3^ Quebec, QC, Canada*

Radiotherapy detectors have advanced and changed over the years and it's important to be aware of their uses and functionality. Optically Stimulated Luminescence Dosimeters (OSLDs) are now used frequently for routine clinical point‐dose measurements because they offer high precision and are easy to use. These dosimeters are versatile, and OSLD have also been used to measure dose in brachytherapy, imaging, and calibration applications. Since the introduction of radiochromic films (RCF) for radiation dosimetry, the scope of RCF dosimetry has expanded steadily to include a wide array of medical applications in radiation therapy and diagnostic radiology. More recently, the multichannel RCF data analysis method has introduced a new standard for external‐beam treatment QA with its high accuracy and efficiency. This presentation will provide comprehensive guidelines on RCF dosimetry for various clinical applications. Radiochromic film is an excellent tool for planar measurements, but comes with its own set of considerations for successful implementation. Plastic scintillators are now available and can be very useful for radiotherapy detection.

**Learning Objectives**:

1. To learn about the clinical use of OSLDs for radiotherapy detection including best uses and drawbacks
2. To outline the procedures to achieve measurement accuracy with an RCF dosimetry system and introduce the paradigm shift to multichannel film dosimetry
3. To describe the use of plastic scintillators in clinical radiotherapy and how to best incorporate this detector in routine clinical practice

S. Kry: Clinical Use of OSLDs

M. Chan: Best Practices with Radiochromic Film

L. Archambault: Adding Plastic Scintillators to the Clinical Physics Toolbox

**Diagnostic Symposium Ballroom C**

***TJC Update and CT Protocol Review***

**MO‐D‐BRC**

## TJC Update and CT Protocol Review

J Webb,^1*^ M Heard^2*^

*The Joint Commission,^1^ Oakbrook Terrace, IL, Sutter Health Support Services,^2^ Sacramento, CA*

CT protocol review has become somewhat of a standard in diagnostic imaging practice. Published guidelines on the process generally require the assistance of a qualified medical physicist (QMP). The process itself contains many inherent challenges, and the QMP is uniquely suited to a leadership role on the CT protocol review team. Additionally, The Joint Commission (TJC) released in January, 2015 its *Revised Requirements for Diagnostic Imaging Services*, to go into effect later in the year. These revised requirements address of CT protocol review and involvement of the QMP. This session will discuss CT protocol review, focused on the challenges and solutions implemented in a consulting environment, and will delve into the philosophy and expectations of the TJC when their new requirements for accreditation go into effect.

**Learning Objectives**:

1. Be introduced to the unique challenges of CT protocol review in a consulting environment
2. Learn some solutions to managing CT protocol review in medical physics consulting
3. Better understand the philosophy of TJC
4. Be introduced to TJC's new Revised Requirements for Diagnostic Imaging Services
5. Understand the expectations of TJC surveyors after their new requirements go into effect

M. Heard: CT Protocol Review in a Consulting Environment

J. Webb: The Joint Commission 2015 Updates

**TUESDAY, MARCH 10**

**Therapy Symposium ‐ SAM Ballroom D**

***Advanced Planning Tools***

**TU‐A‐BRD**

## Advanced Planning Tools

Q Wu,^1*^ L Olsen,^2*^ P Xia^3*^

*Duke University Medical Center,^1^ Durham, NC, Washington University School of Medicine,^2^ St. Louis, MO, The Cleveland Clinic Foundation,^3^ Cleveland, OH*

Treatment planning is an indispensable process for radiation therapy treatment, but there can be significant variability in plan quality. Recently, there is rising interest in designing tools to improve plan quality consistency and plan efficiency by using more automation and less dependence on a planner's experience. This session will introduce both knowledge‐based planning and auto‐planning techniques. Knowledge‐based planning extracts past clinical planning experience to build mathematical models. We will describe the fundamental physics and informatics tools, and discuss the importance of model training and validation. The process and benefits of knowledge‐based treatment planning will be shown with clinical examples. Auto‐planning incorporates many manual planning steps in an automatic routine to achieve the established dosimetric objectives for each specific cancer type. The advantages of this technique will also be discussed.

**Learning Objectives**:

1. To understand knowledge guided planning background: including extracting human planning knowledge into features, machine learning, and modeling techniques
2. To learn how to implement knowledge‐based planning techniques into clinical practice and to recognize the importance of model training and validation, including data size, data quality, modeling parameter statistics, and outlier analysis
3. To describe the underlining principle of the auto‐planning module in a planning system
4. To learn how to use auto‐planning tools and knowledge‐based planning tools to improve plan quality and efficiency through clinical examples

L. Olsen: Clinical Use of Knowledge‐Based Planning

P. Xia: Automated Treatment Planning to Improve Plan Quality

Q. Wu (Duke University Medical Center): The Promise and Appropriateness of Knowledge‐Guided Planning

**Diagnostic Symposium ‐ SAM *Ballroom C***

***Digital Radiography QC***

**TU‐A‐BRC**

## Digital Radiography QC

S Shepard,^1*^ K Hulme^2*^

*UT MD Anderson Cancer Center,^1^ Houston, TX, The Cleveland Clinic,^2^ Cleveland, OH*

CT protocol review has become somewhat of a standard in diagnostic imaging practice. Published guidelines on the process generally require the assistance of a qualified medical physicist (QMP). The process itself contains many inherent challenges, and the QMP is uniquely suited to a leadership role on the CT protocol review team. Additionally, The Joint Commission (TJC) released in January, 2015 its *Revised Requirements for Diagnostic Imaging Services*, to go into effect later in the year. These revised requirements address CT protocol review and involvement of the QMP. This session will discuss CT protocol review, focused on the challenges and solutions implemented in a consulting environment, and will delve into the philosophy and expectations of the TJC when their new requirements for accreditation go into effect.

**Learning Objectives**:

1. Be introduced to the unique challenges of CT protocol review in a consulting environment
2. Learn some solutions to managing CT protocol review in medical physics consulting
3. Better understand the philosophy of TJC
4. Be introduced to TJC's new Revised Requirements for Diagnostic Imaging Services
5. Understand the expectations of TJC surveyors after their new requirements go into effect.

S. Shepard: Practical Issues Surrounding Exposure Indices in Digital Radiography

K. Hulme: Implementation of a Large‐Scale DR QC Program

**Professional Symposium Ballroom D**

***Professional Economics***

**TU‐B‐BRD**

## Professional Economics

B Dirksen,^1*^ G White^2*^

*Mercy Medical Center,^1^ Coralville, IA, Colorado Associates in Medical Physics,^2^ Colorado Springs, CO*

The purpose is to introduce attendees to the health‐care reimbursement system and how it applies to the clinical work of a medical physicist. This will include general information about the different categories of payers and payees, how work is described by CPT codes, and how various payers set values for this work in different clinical settings. We will discuss practical issues related to coding and describing medical physics and other work activities in Radiation Oncology. 2015 is a year of significant changes to the payment system. Many CPT codes have been deleted and replaced with new CPT codes. These codes define some of the most common work performed in our clinics, including treatment planning and delivery. This presentation will describe what work is encompassed in these codes and will give attendees an overview of the changes for 2015 as they apply to radiation oncology. Topics include the impact of the upcoming CMS rule publication schedule changes, typical descriptions of work, estimated work times, presence/supervision requirements, documentation models, and the role of the medical physicist in coding/billing. Broader issues, such as the role of coding/billing consultants, hospital cost/charge ratios and charge master construction, and future impacts of episode of care payments, bundling, medical physicist assistants, and fixes for SGR, will also be discussed. Attendees will have a better understanding of the CPT code changes in 2015. Associated issues from supervision and documentation to billing are discussed. Coding changes are providing challenges to the clinical medical physicist and associated medical facilities. A better understanding of the reimbursement methods will allow the membership to determine how best to adapt with the evolving reimbursement changes.

**Learning Objectives**:

1. Better understand coding changes for 2015
2. Role of billing for consultants and hospital based practices
3. Bundling and its impact on reimbursement

B. Dirksen: 2015 Economics Update

G. White: Coding and Billing Principles and Nuts and Bolts

**Professional Symposium Ballroom C**

***Diagnostic Professional Session***

**TU‐B‐BRC**

## Diagnostic Professional Session

D Gress,^1*^ D Pfeiffer^2*^

*MD Anderson Cancer Center,^1^ Houston, TX, Boulder Community Health,^2^ Boulder, CO*

For a variety of reasons, the diagnostic imaging contingent of AAPM has had a more difficult time trying to estimate workforce requirements than their therapy counterparts. Over the past several years, the Diagnostic Work and Workforce Study Subcommittee (DWWSS) has collected survey data from AAPM members, but the data have been very difficult to interpret. The DWWSS has reached out to include more AAPM volunteers to create a more full and accurate representation of actual clinical practice models on the subcommittee. Though much work remains, through hours of discussion and brainstorming the DWWSS has somewhat of a clear path forward. This talk will provide attendees with an update on the efforts of the subcommittee.

**Learning Objectives**:

1. Learn relevant historical information on this subject
2. Understand the process of the DWWSS in 2014
3. Understand the intended path forward for the DWWSS

The ACR Diagnostic Imaging Centers of Excellence Program takes ACR accreditation in advanced imaging modalities to the next level by providing a comprehensive assessment of the entire medical imaging enterprise, including structure and outcomes. The survey process includes a site visit from a survey team, including a medical physicist.

**Learning Objectives**:

1. Understand the eligibility criteria of the DICOE program
2. Review the survey process
3. Receive practical tips for a successful survey from the perspective of a medical physicist

D. Gress: Diagnostic Workforce Update

D. Pfeiffer: Preparing for an ACR DICOE Site Visit

